# New concepts for an old problem: the diagnosis of endometrial hyperplasia

**DOI:** 10.1093/humupd/dmw042

**Published:** 2016-12-05

**Authors:** Peter A. Sanderson, Hilary O.D. Critchley, Alistair R.W. Williams, Mark J. Arends, Philippa T.K. Saunders

**Affiliations:** 1 MRC Centre for Inflammation Research, The University of Edinburgh, The Queen's Medical Research Institute, 47 Little France Crescent, EdinburghEH16 4TJ, UK; 2 MRC Centre for Reproductive Health, The University of Edinburgh, The Queen's Medical Research Institute, 47 Little France Crescent, EdinburghEH16 4TJ, UK; 3 Division of Pathology, The Royal Infirmary of Edinburgh, 51 Little France Crescent, EdinburghEH16 4SA, UK; 4 Division of Pathology, Edinburgh Cancer Research Centre, Western General Hospital, Crewe Road South, EdinburghEH4 2XR, UK; 5 Centre for Comparative Pathology, The University of Edinburgh, Easter Bush, MidlothianEH25 9RG, UK

**Keywords:** biomarkers, endometrioid endometrial cancer, endometrial hyperplasia, endometrial intraepithelial neoplasia, progression, genomic classification, immunohistochemistry, patient stratification, personalized medicine

## Abstract

**BACKGROUND:**

Endometrial hyperplasia (EH) is a uterine pathology representing a spectrum of morphological endometrial alterations. It is predominantly characterized by an increase in the endometrial gland-to-stroma ratio when compared to normal proliferative endometrium. The clinical significance of EH lies in the associated risk of progression to endometrioid endometrial cancer (EC) and ‘atypical’ forms of EH are regarded as premalignant lesions. Traditional histopathological classification systems for EH exhibit wide and varying degrees of diagnostic reproducibility and, as a consequence, standardized patient management can be challenging.

**OBJECTIVE AND RATIONALE:**

EC is the most common gynaecological malignancy in developed countries. The incidence of EC is rising, with alarming increases described in the 40–44-year-old age group. This review appraises the current EH classification systems used to stratify women at risk of malignant progression to EC. In addition, we summarize the evidence base regarding the use of immunohistochemical biomarkers for EH and discuss an emerging role for genomic analysis.

**SEARCH METHODS:**

PubMed, Medline and the Cochrane Database were searched for original peer-reviewed primary and review articles, from January 2000 to January 2016. The following search terms were used: ‘endometrial hyperplasia’, ‘endometrial intraepithelial neoplasia’, ‘atypical hyperplasia’, ‘complex atypical hyperplasia’, ‘biomarker’, ‘immunohistochemistry’, ‘progression’, ‘genomic’, ‘classification’ and ‘stratification’.

**OUTCOMES:**

Recent changes to EH classification reflect our current understanding of the genesis of endometrioid ECs. The concept of endometrial intraepithelial neoplasia (EIN) as a mutationally activated, monoclonal pre-malignancy represents a fundamental shift from the previously held notion that unopposed oestrogenic stimulation causes ever-increasing hyperplastic proliferation, with accumulating cytological atypia that imperceptibly leads to the development of endometrioid EC. Our review highlights several key biomarker candidates that have been described as both diagnostic tools for EH and markers of progression to EC. We propose that, moving forwards, a ‘panel’ approach of combinations of the immunohistochemical biomarkers described in this review may be more informative since no single candidate can currently fill the entire role.

**WIDER IMPLICATIONS:**

EC has historically been considered a predominantly postmenopausal disease. Owing in part to the current unprecedented rates of obesity, we are starting to see signs of a shift towards a rising incidence of EC amongst pre- and peri-menopausal woman. This creates unique challenges both diagnostically and therapeutically. Furthering our understanding of the premalignant stages of EC development will allow us to pursue earlier diagnosis and facilitate appropriate stratification of women at risk of developing EC, permitting timely and appropriate therapeutic interventions.

## Introduction

Endometrial cancer (EC) is the most common gynaecological malignancy affecting women in developed countries and the second most common gynaecological malignancy world-wide, due to the higher rates of cervical cancer in the developing world (Ferlay *et al*., 2015). The incidence of EC is steadily increasing, largely owing to an ageing population and escalating rates of obesity (Renehan *et al*., 2010; Ferlay *et al*., 2015; Wise *et al*., 2016). In spite of the frequency of this disease, awareness amongst the general population is low and EC research is somewhat underfunded relative to its societal burden (Carter and Nguyen, 2012). If diagnosed and treated at Stage I or II (International Federation of Gynaecology and Obstetrics [FIGO] stages), EC 5-year survival figures stand at ~92% and 75%, respectively (Creasman *et al*., 2006; Murali *et al*., 2014). Women diagnosed with advanced EC, FIGO stages III and IV, have 5-year survival figures reported at 57–66% and 20–26%, respectively (Murali *et al*., 2014).

ECs have classically been described *via* a dualistic model which divides them into ‘Type 1’ and ‘Type 2’ carcinomas, based upon histological, clinical and metabolic features (Bokhman, 1983). The Type 1 carcinomas, of which the endometrioid histological subtype accounts for ~75%, typically represent low-grade tumours which are often amenable to surgical treatment (Silverberg *et al*., 2003; Murali *et al*., 2014). Type 1 ECs are considered oestrogen-dependent and are frequently associated with hyperplastic proliferation of the endometrial glands; they are characteristically seen in postmenopausal obese women. In our own studies, we have demonstrated differential expression of oestrogen receptors alpha and beta in Type 1 ECs according to tumour grade (Collins *et al*., 2009).

Conversely, Type 2 ECs tend to be oestrogen-independent and include the clinically aggressive ‘serous’ and ‘clear cell’ histological subtypes. Type 2 ECs are more often associated with endometrial atrophy in the postmenopausal woman rather than with endometrial hyperplasia (EH) as in Type 1 EC. They are linked with a much poorer clinical prognosis (Creasman *et al*., 2006; Abu-Rustum *et al*., 2010; Matias-Guiu and Prat, 2013). Despite the seemingly intuitive division of ECs into these two types, this classification is far from perfect. There is a significant overlap between Type 1 and Type 2 ECs. For example, 10–19% of endometrioid ECs are deemed high-grade and have clinical, histopathological and molecular features that are more akin to Type 2 ECs (Voss *et al*., 2012; Brinton *et al*., 2013). Mixed histological patterns incorporating endometrioid and serous morphology also exist (Mackenzie *et al*., 2015).

Large-scale, next-generation sequencing projects and advanced molecular pathology methodologies are spearheading an expansion of personalized medicine within cancer care. Notably ‘pre-cancer’ detection and patient risk stratification are increasingly important for early diagnosis and prevention of cancer (Berman *et al*., 2006). There are several lines of evidence that a diagnosis of EH may precede the development of endometrioid EC and that the two share common predisposing risk factors (Table I). The incidence of EH is roughly three times higher than EC and certain atypical forms of EH are considered to represent direct precursor lesions to endometrioid EC (Reed *et al*., 2009; Ellenson *et al*., 2011). In the current literature, two main classification systems have been used to subdivide EH. The more recent of the two places greater emphasis on robust diagnostic reproducibility and develops the entity endometrial intraepithelial neoplasia (EIN) as a premalignant lesion with significant oncogenic potential (Mutter, 2000a).

**Table I:** Risk factors for the development of endometrial hyperplasia (EH).

Risk factor category	Risk factor
Non-modifiable	Age >35 years Caucasian ethnicity Family history
Menstrual	Postmenopausal status Early menarche/late menopause Prolonged perimenopause Null parity
Co-morbid conditions	Obesity Diabetes mellitus Polycystic ovarian syndrome (PCOS) Functional tumours, e.g. granulosa cell Lynch syndrome/hereditary non-polyposiscolorectal cancer (HNPCC)
Iatrogenic	Long-term Tamoxifen therapy Oestrogen only hormone replacement therapy(HRT) Exogenous oestrogen exposure
Others	Smoking Genetic mutations

As we will explore in this review, classifying EHs can be troublesome, largely due to the heterogeneity of EH lesions. This makes the task of stratifying women at risk of progression to endometrioid EC all the more challenging. We aim to appraise the current EH classification systems used for patient risk stratification. In addition, we summarize the evidence base regarding the use of immunohistochemical biomarkers for EH and discuss the future potential of genomic analysis.

## Methods

PubMed, Ovid^®^ Medline and the Cochrane Collaborative database were searched for high-quality, peer-reviewed primary papers and review articles, from January 2000 to January 2016. Using Boolean operators, several search variants using the following keyword terms were combined: ‘endometrial hyperplasia’, ‘endometrial intraepithelial neoplasia’, ‘atypical hyperplasia’, ‘complex atypical hyperplasia’, ‘biomarker’, ‘immunohistochemistry’, ‘progression’, ‘genomic’, ‘classification’ and ‘stratification’. We reviewed all identified manuscripts and included them, where appropriate, in the scope of this review. The reference lists of included manuscripts were also searched for any older, relevant sources and included where appropriate. In addition, a hand-search identified several pertinent websites, guidelines and policy documents that were included. Non-English language texts were excluded.

## Endometrial hyperplasia

**Figure 1:**
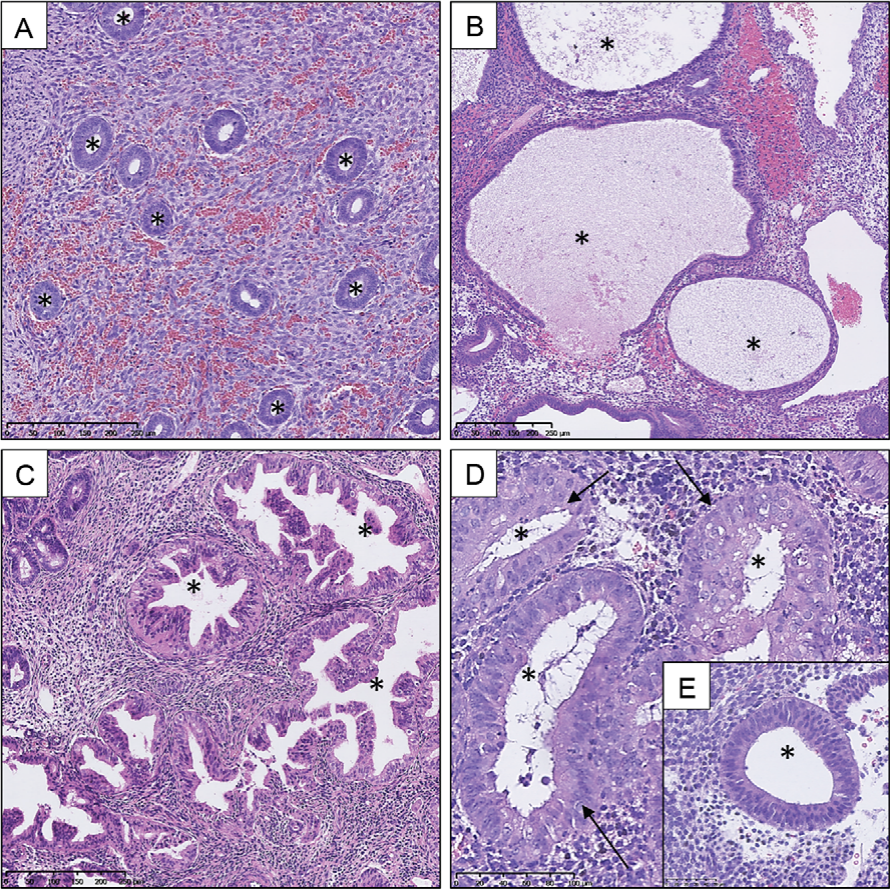
Haematoxylin and eosin (H&E) stained sections demonstrating variation in size and shape of endometrial glands within a spectrum of endometrial hyperplasia (EH) lesions compared to proliferative endometrium (PE). Selection of glands marked by * in lumen. (**A**) PE, (**B**) hyperplasia without atypia: large cystically dilated glands, (**C**) endometrial intraepithelial neoplasia (EIN): varied and irregular gland morphology, (**D**) high-power EIN lesion: cytological atypia within glands (arrow) and (**E**) excerpt of a phenotypically ‘normal’ gland cytology within the same section as D for comparison. Varying magnifications: see scale bars.

EH represents a spectrum of irregular morphological alterations, whereby abnormal proliferation of the endometrial glands results in an increase in gland-to-stroma ratio when compared to endometrium from the proliferative phase of the cycle (Ellenson *et al*., 2011; Kurman *et al*., 2014). The proliferating glands in EH can vary greatly in size and shape, and cytological atypia may be present (Fig. 1). Historically, several different terms have been employed to describe this abnormal proliferation of the endometrium, including: ‘adenomatous hyperplasia’, ‘atypical hyperplasia’ and ‘carcinoma-in-situ’ (Giuntoli *et al*., 2014). In developed countries, there are an estimated 200 000 new cases of EH per annum (Ozdegirmenci *et al*., 2011). However, this is likely an underestimation since epidemiological registry data on EH patients can differ significantly between institutions.

**Figure 2:**
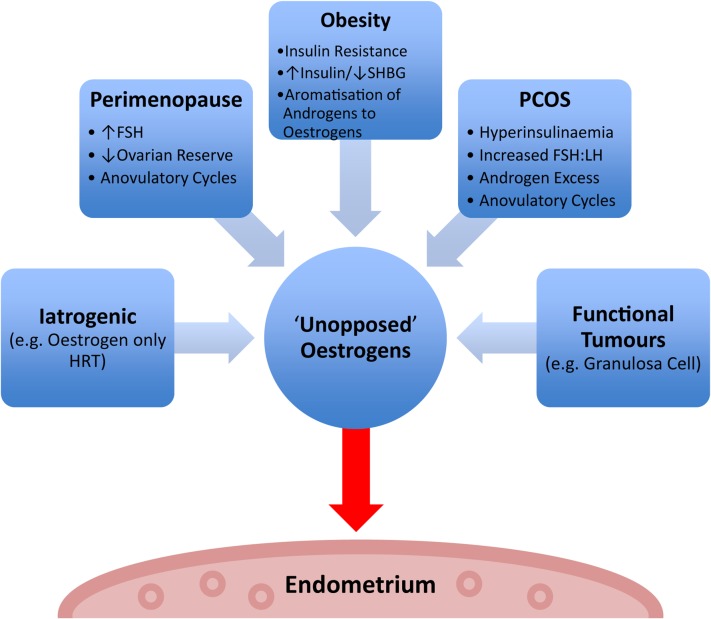
Factors contributing to ‘unopposed’ oestrogen stimulation of the endometrium. SHBG = sex hormone binding globulin, FSH = follicle stimulating hormone, FSH:LH = follicle stimulating hormone to luteinizing hormone ratio, HRT = hormone replacement therapy, PCOS = polycystic ovarian syndrome.

It is presumed that most EHs develop in a background of chronic stimulation of the endometrium by oestrogens unopposed by a progestin, occurring secondary to a number of possible conditions (Trimble *et al*., 2012) (Fig. 2). The majority of women with EH will present clinically with abnormal uterine bleeding (AUB) and EHs have previously been estimated to account for 15% of all cases of postmenopausal bleeding (Lidor *et al*., 1986). The main risk factors for the development of EH (Table I) are similar to those associated with EC. Two particularly high-risk patient populations are (i) obese peri/postmenopausal women, owing in part to peripheral aromatization of androgens to oestrogens in adipose tissue, coupled with erratic anovulatory cycles and (ii) premenopausal patients with polycystic ovarian syndrome (PCOS), due to hyperandrogenic anovulation. Although stimulation of the endometrium by oestrogens is considered the main risk factor for developing EH, other causes such as immunosuppression have been suggested (Bobrowska *et al*., 2006). A retrospective study of 45 immunosuppressed renal transplant recipients with AUB found a ∼2-fold increase in the incidence of EH (69% *versus* 34%) compared to non-transplanted immunocompetent controls with AUB (Bobrowska *et al*., 2006).

The clinical importance of a diagnosis of EH relates to the long-term risk of progression to endometrioid EC and it is generally accepted that cytological atypia is the principal histological characteristic when assessing EHs for malignant potential (Ellenson *et al*., 2011). However, not all EHs will progress to malignancy; some EHs occur secondary to oestrogenic proliferation without an underlying malignant mechanism. These patients may be asymptomatic and in some cases the EH may regress without ever being detected.

Analysis and classification of EH is not without challenge. Firstly, the endometrium is a dynamic, multicellular tissue structure that undergoes hormonally driven cyclical proliferation, shedding and rapid healing. In premenopausal women, this renders a consistently ‘normal’ or ‘control’ state difficult to establish (Ellenson *et al*., 2011). This is especially challenging in peri-menopausal women who will often have erratic menstrual cycles. Secondly, EHs can be very heterogeneous and may present as focal or diffuse lesions, often with multifaceted architectural and cytological features. An EH lesion may be shed with menses, may be entirely removed or under-sampled with a diagnostic biopsy, or may regress with progestin treatment or even spontaneously without intervention (Allison *et al*., 2008; Trimble *et al*., 2012). As such, classifying EHs into clinically meaningful groups that permit correlation with potential for malignant transformation can be fraught with difficulty.

Several histological classification methods have been proposed aiming to correlate EH architecture and cytological features with the risk of progression to endometrioid EC (Chandra *et al*., 2016). The two prominent classification systems are (i) The World Health Organisation (WHO) system, established in 1994 with revision in 2003, which is widely known within current clinical gynaecological practice and (ii) The endometrial intraepithelial neoplasia (EIN) system, introduced in 2000 (Mutter, 2000a) and was endorsed in 2014 by the WHO as part of their most recent classification of tumours of the female reproductive organs (Kurman *et al*., 2014).

### The World Health Organization (WHO) 1994 classification system

Multiple pathological classification systems, stretching back to 1963, have been used to describe EH (reviewed in Chandra *et al*., 2016). Each system struggles in some part to describe the spectrum of heterogeneity observed between individual EH lesions and correlate this with clinical management.

In 1994, the WHO recommended a classification system based upon the histological features of EH lesions, in an attempt to stratify EHs based on their potential for malignant transformation (Scully *et al*., 1994). The system focused on the glandular/stromal architectural pattern of the endometrium and the presence or absence of cytological atypia. Four groups emerged and are detailed in Table II, although the simple atypical hyperplasia group is very rarely seen and some would question its reproducibility and clinical relevance as a category (Kendall *et al*., 1998; Bergeron *et al*., 1999).

**Table II:** The World Health Organisation 1994 classification of EH (Palmer *et al*. 2008; Ellenson *et al*. 2011; Chandra *et al*., 2016).

WHO94 categories	Histological and cytological features
Simple hyperplasiawithout atypia (SH)	Irregularly shaped and sized glands Cystic dilatation Abundant cellular stroma No back to back crowding Nuclear pseudo-stratified glands but nonuclear atypia Variable mitotic activity
Simple atypical hyperplasia(SAH)	As per SH including nuclear atypia
Complex hyperplasiawithout atypia (CH)	Crowded glands—can be complex ortubular, with or without dilatation Sparse intervening stroma Oval, bland nuclei with uniform shape Variable mitotic activity
Complex atypicalhyperplasia (CAH)	Tightly packed glands Very little intervening stroma Nuclear atypia

Nuclear atypia = enlarged and rounded nuclei, irregular, clumped chromatin, thickened nuclear membrane and prominent nucleoli.

These four groups appeared to correlate with long-term follow-up studies of patients diagnosed with EH that had been conducted previously (Kurman *et al*., 1985; Ferenczy and Gelfand, 1989). Arguably, the most influential EH follow-up study was reported in 1985 by Kurman *et al*. (1985). In this study, the authors performed a retrospective analysis of 170 ‘untreated’ EH patients who had been diagnosed with EH on uterine curettage. The mean follow-up period for the women was 13.4 years, during which time a hysterectomy was not performed <1 year following the index diagnosis. Of the 170 women in the study, 13 progressed to EC during the follow-up period (Kurman *et al*., 1985). The authors published EC progression rates of 1% (simple hyperplasia, SH, without atypia), 3% (complex hyperplasia, CH, without atypia), 8% (simple atypical hyperplasia, SAH) and 29% (complex atypical hyperplasia, CAH), respectively, for the four categories (Kurman *et al*., 1985). However, the differences in progression between the four categories were not statistically significant and given the small number of cancer patients and a lack of controls, there is difficulty extrapolating a true rate of progression (Lacey *et al*., 2008a; Ellenson *et al*., 2011).

Lacey *et al*. conducted a nested case–control study in 2007. The authors analysed 138 cases of EH (and 241 matched controls) who progressed to EC at least 1 year following an index EH diagnosis. They demonstrated a 40% probability of developing EC following a diagnosis of atypical hyperplasia (incorporating both simple and complex variants), compared to a 10% probability when atypia was not present (Lacey *et al*., 2008a). Lacey *et al*. (2008a) commented on the need to increase sensitivity and specificity when diagnosing atypical hyperplasia and to find methods of identifying the rare non-atypical EH lesions that are also likely to progress to EC.

When first introduced, the WHO94 system was considered an improved approach to EH classification since it correlated the histological features of EH lesions with clinical outcome data (Baak and Mutter, 2005). However, the subjective nature of this system has meant that significant diagnostic variation occurs between pathologists and overall reproducibility is poor (Skov *et al*., 1997; Kendall *et al*., 1998). Cytological atypia is not always uniformly seen in individual EH samples and the use of subjective atypia grading scales, i.e. mild, moderate and severe by individuals has been problematic, especially when translating the scheme to clinical management (Kendall *et al*., 1998). These issues were highlighted by Trimble *et al*. in a study in which 289 endometrial specimens with a community diagnosis of atypical EH were re-reviewed by specialized gynaecological pathologists using WHO94 criteria; 25% of cases were downgraded to a less severe histology than atypical EH and 29% were upgraded to EC (Trimble *et al*., 2006).

Another difficulty identified within the WHO94 system is the relationship between the diagnostic groups and clinical treatments. The four-tier WHO94 system does not straightforwardly correspond to the separate therapeutic options available (i.e. surgical, medical or observational) (Baak and Mutter, 2005), which may contribute to a tendency for surgical overtreatment due to the fear of malignant progression for lesions with no underlying sinister mechanism (Baak *et al*., 2001).

Clinical treatment based upon WHO94 classification of EH varies between institutions, with patient-specific factors, i.e. age, co-morbid status and future fertility wishes, influencing decision-making. EHs without nuclear atypia have been documented to regress back to normal endometrium in around 90% of cases, with no progression to malignancy but a recurrence rate of ~10% (Hannemann *et al*., 2010). When atypia is seen, definitive surgical treatment in the form of a total hysterectomy is normally offered, since the risk of progression to endometrioid EC is so much higher. Furthermore, there is the risk of a concurrent EC already being present in the uterus that may have gone undetected on biopsy.

### The endometrial intraepithelial neoplasia (EIN) 2000 classification system

As reviewed above, it is well established that nuclear atypia within hyperplastic lesions confers the highest risk of progression to endometrioid EC (Sherman and Brown, 1979; Kurman *et al*., 1985). Research in the 1980s, spearheaded by Jan Baak, developed a prognostic tool designed to predict EC risk based upon morphometric analysis of the nuclear features within EH lesions (Ausems *et al*., 1985). It was subsequently found that, through a combined analysis of nuclear and architectural features, the prognostic value of morphometric analysis could be increased (Baak *et al*., 1988). This work culminated in the development of a weighted likelihood ratio called the ‘*D*-score’. The *D*-score centres on three key EH features: (i) volume percentage of stroma, (ii) outer surface density of the glands and (iii) the standard deviation, SD of the shortest nuclear axis within glandular cells (Baak *et al*., 1988; Baak and Mutter, 2005). The following equation is used:

*D*-score = 0.6229 + 0.0439 × (volume percentage stroma) − 3.9934 × natural logarithm, Ln (SD shortest nuclear axis) − 0.1592 × (glands outer surface density) (Baak *et al*., 1988).

By applying the *D*-score, hyperplastic biopsies with a score of ≤1 have a high rate of progression to EC, whereas biopsies with a score of >1 almost never progress to EC (Baak *et al*., 1992, 2001). In addition, this system has been shown to be highly reproducible (Baak and Mutter, 2005). Advances in molecular genetics, occurring at around a similar time as the progress being made with morphometric analysis, recognized a shared monoclonal pattern of development between atypical hyperplastic lesions and ECs (Jovanovic *et al*., 1996). These findings would be consistent with mutated cells stemming from a common progenitor, proliferating more rapidly than their neighbours and resulting in clonal expansions of aberrant cells detected as lesions (Jovanovic *et al*., 1996).

A multicentre European study was conducted in 1999 by Bergeron *et al*. to investigate and assess both intra- and inter-observer variability in the diagnosis of 56 endometrial samples using the WHO94 classification system (Bergeron *et al*., 1999). The investigators noted significant disagreement in the diagnoses of CH and atypical hyperplasia between pathologists (Bergeron *et al*., 1999). They concluded that histological classification should be simplified into two groups; a combined category for SH and CH, referred to as ‘hyperplasia’, and a combined category for atypical hyperplasia and well-differentiated adenocarcinoma, called ‘endometrial neoplasia’. The rationale for this being that by utilizing two groups, one benign and one neoplastic, reproducibility would be increased and a two-tier system would align easily with therapeutic interventions, i.e. medical or surgical (Bergeron *et al*., 1999).

Acknowledging the deficiencies within the WHO94 system, in 2000 the Endometrial Collaborative Group introduced the notion of EIN, as part of a newer classification system (Mutter, 2000a). The EIN concept incorporated advances in morphometric understanding and recognized the novel molecular research occurring in the field of endometrial precancers at that time (Jovanovic *et al*., 1996; Mutter *et al*., 1996; Mutter, 2000a). The EIN classification system divides hyperplastic endometrial lesions into two groups: (i) benign EH and (ii) EIN. This is based on objective diagnostic criteria (Table III) that can be determined from a haematoxylin and eosin (H&E) stained endometrial section. In essence, these criteria emulate what the *D*-score achieves; however, they can be ascertained quickly by a pathologist using routine light microscopy (Owings and Quick, 2014).

**Table III:** Haematoxylin and eosin section diagnostic criteria for endometrial intraepithelial neoplasia (EIN).

EIN criterion	Comments
Architecture	Area of glands exceeds that of stroma (VPS < 55%)
Cytology	Cytology differs between architecturally crowdedfocus and background
Diameter >1 mm	Maximum linear dimension of the lesion exceeds1 mm
Exclude mimics	Benign conditions with overlapping criteria: basalis,secretory, polyps, repair, etc.
Exclude cancer	Carcinoma if maze-like meandering glands, solidareas or appreciable cribriforming

NB: All criteria must be met in order for a diagnosis of EIN to me made.VPS = volume percentage stroma.Reproduced with permission from Baak and Mutter (2005).

**Figure 3:**
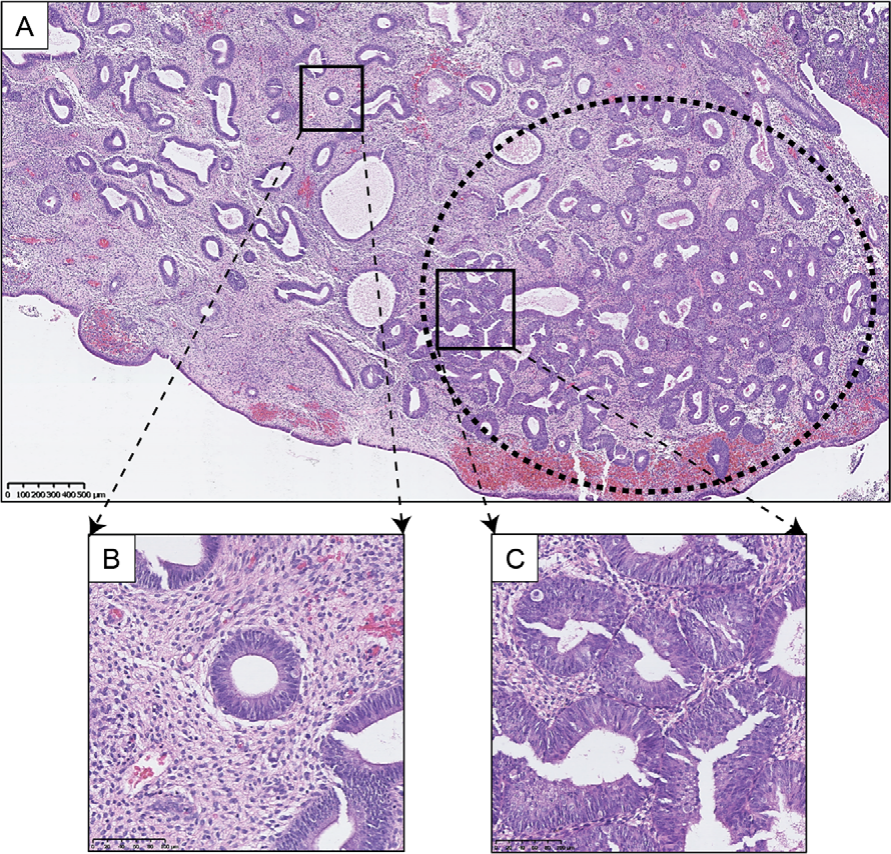
Clonal expansion of EIN. H&E staining of an endometrial biopsy. (**A**) Low power view of a clonal expansion of EIN, with prominent gland crowding (marked in oval with bold dashes), in a background endometrium demonstrating benign EH, (**B**) high-power view of background endometrium, (**C**) high-power view of EIN lesion glands. Varying magnifications: see scale bars.

EIN lesions are defined as monoclonal proliferations of architecturally and cytologically altered premalignant endometrial glands, which are prone to transformation to endometrioid EC (Mutter, 2000a) (Fig. 3). Prior to the inception of EIN, a general belief was held that unopposed oestrogenic stimulation caused ever-increasing endometrial proliferation, with accumulating cytological atypia that imperceptibly led to the development of endometrioid EC. The EIN mechanism proposes that genetic alterations within the endometrium initially occur at a level undetectable by light microscopy. These ‘latent’ genetically transformed cells can be present for numerous years within cycling endometrium (Mutter *et al*., 2007). Through the ongoing accrual of genetic damage, higher risk mutated clones assert themselves phenotypically, exhibiting architectural and cytological characteristics that are indicative of EIN (Table III). The mutant clones are subject to endocrine modifiers, with oestrogens acting as promoters and progestins (natural or synthetic) acting as suppressors. Variations in the balance of endocrine modifiers can alter the balance of progression to EC *versus* hyperplastic lesion involution (Mutter *et al*., 2007). In contrast, benign EH lesions are deemed diffuse and polyclonal, occurring globally due to an unopposed oestrogenic stimulus (Mutter, 2000a; Baak and Mutter, 2005). Crucially, these lesions do not exhibit cytological differences between architecturally crowded and uncrowded glandular regions; their appearance at any one time point is entirely dependent on the predisposing hormonal milieu (Mutter *et al*., 2007).

The EIN concept recognizes the importance of unopposed oestrogenic stimulation; however, it distinguishes it from the separate event of a mutationally activated clone developing an oestrogenic background (Mutter, 2000b; Mutter *et al*., 2007; Owings and Quick, 2014). Histologically, this can prove difficult to segregate, as early lesions can have a combination of appearances that may include the clone (EIN) within an oestrogen stimulated tissue background. The idea of separating the two events, mutational activation and oestrogenic promotion, not only permits examination of the two components separately, but gives a more comprehensible model of the multistep carcinogenic process that is similar to that described in many other tissue types (Vineis *et al*., 2010).

The EIN classification system has been shown to be reproducible between observers and straightforward to establish in standard pathological practice (Kane and Hecht, 2012; Usubutun *et al*., 2012). Hecht *et al*. analysed the use of the *D*-score compared to EIN criteria in their 2005 retrospective study of 97 EH biopsies. They demonstrated that subjective EIN assessment correlates well with objective morphometric analysis. All EHs that progressed to EC occurred in patients whose endometrial biopsies were deemed high risk by both methods, although interestingly 15 samples were given a *D*-score of ≤1 (i.e. high risk) and yet were subjectively classified as non-EIN (Hecht *et al*., 2005).

**Figure 4:**
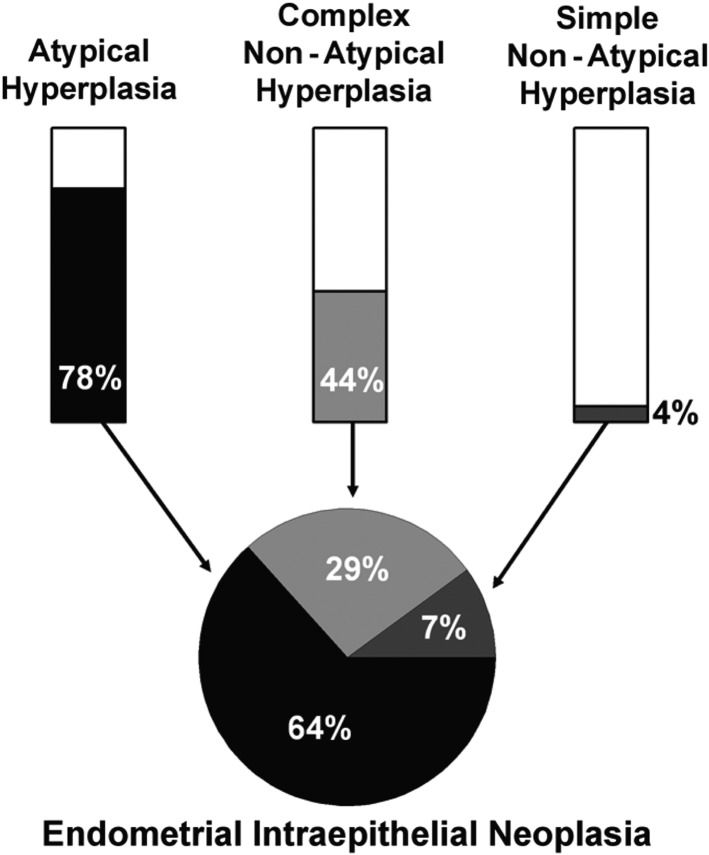
Correlation of WHO and EIN diagnoses (Hecht *et al*., 2005). (1) The bar graphs show the approximate percentage of each WHO94 category that would be considered as EIN. Residual WHO94 EHs that are not diagnostic of EIN (i.e. the white areas of the bars) are attributed to unopposed oestrogen (anovulatory cycles), polyps and other causes. (2) The pie chart demonstrates the relative contributions of each hyperplasia subtype to the EIN diagnostic category in a series of 97 cases with 28 EIN examples by Hecht *et al*. from their 2005 study. Republished with permission from Hecht *et al*. (2005).

EIN classification categories do not correspond directly to specific categories in the WHO94 system (Mutter, 2000b), although, there is an element of recognizable overlap. Most SH and some CHs will align into the benign EH category and many CHs and most CAHs will align into the EIN category. A useful visual summary was provided by Hecht and colleagues and this is reproduced, with permission, in Fig. 4 (Hecht *et al*., 2005). Both the EIN and WHO94 systems are governed by different diagnostic elements and so the two systems are not directly comparable (Mutter *et al*., 2007).

### Endorsement of the EIN system: the WHO 2014 classification system

**Figure 5:**
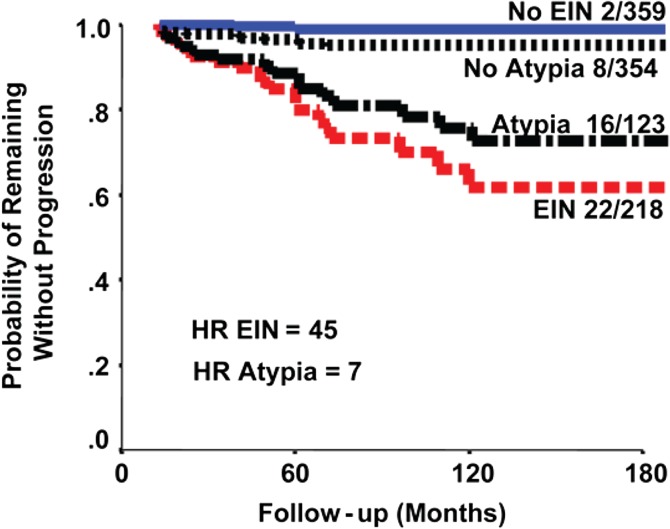
Data to suggest that EIN classification system more accurately predicts progression to EC than the WHO94 system (Baak *et al*., 2005a). Patients with at least 1-year follow-up. The graph compares the WHO94 ‘Atypia’ and EIN systems in terms of prognostic accuracy. The study reported that the EIN classification is superior to the WHO94 classification for discerning cases at risk of progression to future EC. The fractions are the number that progressed to cancer over the total in that subgroup. HR = hazard ratio. Republished with permission from Baak *et al*. (2005a).

The EIN system has several proposed clinic-pathological advantages over the WHO94 system, most notably diagnostic reproducibility and correlation with clinical management. Clinical outcome data suggest that ~40% of women diagnosed with EIN will have an EC diagnosed within 12 months of index biopsy (Baak *et al*., 2005a; Mutter *et al*., 2008). The mostly likely explanation for this is the presence of a concurrent EC that was not sampled on initial biopsy. Those women who do not develop EC within 12 months are 45 times more likely to develop a future EC (Baak *et al*., 2005a). Baak *et al*. (2005a) also argued that the EIN classification system more accurately predicts progression to EC than the WHO94 system (Fig. 5, reproduced with permission). A later study reported that both EIN and atypical hyperplasia have similar risks of progression to EC when followed-up for 12 months after the index diagnosis (Lacey *et al*., 2008b). It is only in the last few years that the EIN classification system has started to gain widespread recognition. This may reflect regional variation in gynaecological and gynae-pathological practices and, until recently, the lack of a standardized approach to the clinical management and surveillance of EH.

In 2014, the WHO published its 4th edition of *Classification of Tumours of the Female Reproductive Organs* (Kurman *et al*., 2014). The new edition clarifies the WHO position on the classification of EH and it endorses the EIN diagnostic system (Table III). WHO 2014 differentiates EHs into two categories: (i) hyperplasia without atypia and (ii) atypical hyperplasia/EIN (both terms synonymous) (Zaino *et al*., 2014; Emons *et al*., 2015). The American College of Obstetricians and Gynecologists (ACOG) released a committee opinion paper detailing EIN in 2015 (Committee on Gynecologic Practice, 2015). It is intended for dissemination to all interested stakeholders regarding the EIN classification method and provides advice on the clinical management of EH and EIN. The ACOG favours the use of EIN terminology over ‘atypical hyperplasia’ providing recognition that these lesions are distinctly neoplastic and harbour significant malignant potential. In the UK, the Royal College of Obstetricians and Gynaecologists (RCOG) released their first guideline for the management of EH in 2016 (Gallos *et al*., 2016). The RCOG refer to ‘atypical hyperplasia’ as the premalignant lesion, although they acknowledge that the term is interchangeable with EIN (Gallos *et al*., 2016). The RCOG guidance goes one step further and includes a management algorithm for EH, detailing preferred treatments and advising on timing of endometrial biopsy for patients undergoing conservative or medical management.

The EIN system offers a robust and reproducible classification method that correlates well with the risk of progression of EH to EC. Subjective histopathological assessment based upon the objective EIN criteria can be undertaken on H&E stained sections of tissue and so a potential role for immunohistochemical biomarkers has been established as a way of further stratifying ‘at risk’ EH patients for malignant progression.

## Immunohistochemical biomarkers for diagnosis of endometrial hyperplasia and predicting progression of endometrial hyperplasia to endometrial cancer

Biomarkers are defined as ‘characteristics that can be objectively measured and evaluated as indicators of normal biological processes, pathogenic processes, or pharmacological responses to a therapeutic intervention’ (Atkinson *et al*., 2001). Several immunohistochemical biomarkers have already been investigated for use as diagnostic adjuncts to H&E staining to: (i) aid diagnosis and classification of EHs; and (ii) predict the likelihood of transition from EH to EC. The optimal molecular biomarker would be one that could both reliably and reproducibly distinguish between normal/benign, premalignant and malignant endometrium, in addition to indicating/predicting transition between these three groups. To date, no single candidate has been found to fulfil this role entirely and so the search continues. In the following sections, we review several promising candidates identified from the literature and appraise their role as biomarkers for EH diagnosis and progression to EC.

### Regulators of steroid action or inflammation

#### Oestrogen receptors alpha and beta

Oestrogens bind to one of two nuclear receptors (Oestrogen receptors alpha and beta, ERα and ERβ), which are both encoded by independent genes (reviewed in Gibson and Saunders, 2012). The full-length receptors classically operate as ligand-dependent transcription factors with subsequent modulation of gene expression. Several immunohistochemical studies have reported nuclear expression of both oestrogen-receptor subtypes (ERs) in the glandular and stromal regions of the normal premenopausal and postmenopausal endometrium, with differences in their distribution described between the phases of the normal cycling endometrium (Lessey *et al*., 1988; Snijders *et al*., 1992; Fujishita *et al*., 1997; Critchley *et al*., 2001). These and other studies have consistently demonstrated the importance of oestrogens in regulating endometrial cell proliferation, angiogenesis and inflammation (Gibson and Saunders, 2012). The causative relationship between excess oestrogen exposure, EH and endometrioid ECs has been unequivocally established and concerns have been expressed about the potential for environmental compounds, classified as endocrine disruptors, to increase the risk of malignant transformation (reviewed in Gibson and Saunders, 2014). Numerous studies have examined ER status of patients diagnosed with malignant endometrial disease, endeavouring to analyse the association between ER status, lesion histology, progression and survival (Geisinger *et al*., 1986; Sutton *et al*., 1989; Chamber *et al*., 1990; Lukes *et al*., 1994; Sivridis *et al*., 2001; Ashton *et al*., 2009; Zannoni *et al*., 2013). These remain active areas of investigation to date.

Our review identified several studies that have compared ER expression between normal endometrium, EH and EC tissues with conflicting findings reported (Supplementary Table SI). For example, Uchikawa *et al*. and Bircan *et al*. both described increased ERα expression within EHs when compared to normal secretory endometrium (Uchikawa *et al*., 2003; Bircan *et al*., 2005). In a normal fertile cycle, ERα expression in the epithelial cells is down-regulated in the secretory phase in response to progesterone driven changes in gene expression (Critchley *et al*., 2001; Gibson and Saunders, 2012), hence sustained expression of ERα may reflect reduced action of progesterone (see below).

Hu *et al*. assessed 114 patient samples (15 normal, 37 ECs, 30 SHs, 13 CHs and 20 atypical hyperplasias) for both ERα and ERβ expression using fixed tissue sections in 2008 (Hu *et al*., 2008). They reported a significant increase in the positivity index (% of stained cells) of ERα in samples of SH and CH compared with proliferative endometrium (PE). In contrast, Chakravarty *et al*. (2008) did not report any difference in the expression levels of either ERα or ERβ between PE and SH in their 2008 study.

Owing to the complex interactions between the cycling endometrium and steroid hormones, it is perhaps not so surprising that there are conflicting literature findings regarding ER expression levels between normal, hyperplastic and malignant endometrial lesions. In Stage I EC, changes in ERα are also reported to be independent of ERβ and loss of expression of ERα is a feature of a more malignant phenotype (Collins *et al*., 2009). Several authors identified in our review have described that lower ERα expression can be found in atypical EH and ECs, implying that loss of receptor expression may occur as the lesions progress (Nunobiki *et al*., 2003; Uchikawa *et al*., 2003; Bircan *et al*., 2005; Hu *et al*., 2008). However, the significance levels differ between the studies and variations in methodology and immunohistochemical scoring have to be taken into account, which all limit the potential utility for EH/EIN.

#### Progesterone receptors

Progesterone is a steroid hormone that is essential to female reproductive function. Primarily produced by the corpus luteum following ovulation, it counteracts the proliferative effects of oestrogen by inducing secretory differentiation of the glandular and stromal compartments of the endometrium and down-regulates ERα (Graham and Clarke, 1997; Deligdisch, 2000). Progesterone asserts its actions *via* progesterone receptors (PR), which are members of the same superfamily of ligand-activated transcription factors as the oestrogen receptors (Graham and Clarke, 1997). There are two main isoforms of PR, namely PR-A and PR-B, which are both encoded by a single *PGR* gene (Gadkar-Sable *et al*., 2005). Extensive studies using *in vitro* cell systems as well as genomic analyses have identified *PR* as an oestrogen regulated gene (reviewed in Diep *et al*., 2016). PR isoforms are spatially and temporally controlled within the endometrial compartments across the menstrual cycle in response to fluctuating concentrations of ovarian steroids (Wang *et al*., 1998; Mote *et al*., 1999; Leyendecker *et al*., 2002).

Chronic exposure to oestrogens unopposed by progesterone is considered a key component in the development of EH and EC (Amant *et al*., 2005). The role of PR has, therefore, been extensively investigated in EC development and progression, with loss of PR being shown to be associated with poor survival and metastatic disease (Kleine *et al*., 1990; Kadar *et al*., 1993; Fukuda *et al*., 1998; Tangen *et al*., 2014). Progestin treatment is used as a medical therapy for women diagnosed with EH with reported regression rates of 89–96% (Gallos *et al*., 2010). For women diagnosed with EIN who wish to preserve their fertility or who are not suitable for surgery, progestins are also recommended as a first line medical therapy (Committee on Gynecologic Practice, 2015; Gallos *et al*., 2016). In addition, intrauterine progestin (e.g. Levonorgestrel, LNG/Mirena^®^ IUS, Bayer, UK) has also been explored as a hormonal treatment for early stage endometrioid EC with varying success reported (Montz *et al*., 2002; Ramirez *et al*., 2004; Dhar *et al*., 2005). The delivery of progestins is challenging due to the short half-life and doses that are required.

This review captured five studies where PR expression was investigated within EH tissues (Supplementary Table SI). Three authors reported reduced trends of expression of PR within EHs compared to control endometrium (Nunobiki *et al*., 2003; Uchikawa *et al*., 2003; Pieczyńska *et al*., 2011). On the contrary, Ghabreau *et al*. (2004) demonstrated a progressive increase in PR expression from non-atypical EH to atypical EH. Orejuela *et al*. reported no significant difference in PR expression between normal endometrium and EHs; they also note a slight reduction in PR expression between ECs compared to EH and normal endometrium groups; however, this did not reach statistical significance (Orejuela *et al*., 2005).

Given the clinical application of progestins as a medical treatment for EH, it is perhaps a little surprising that studies investigating PR expression in EH tissues do not reach an agreement regarding its utility as either a diagnostic EH tool or marker of progression to EH. However, as is seen with ER expression, the interplay between the endometrium and steroid hormones and their co-receptors make any potential changes in PR expression pattern difficult to interpret. PR expression may be of novel use as a method of predicting response to progestin therapy in the treatment of EH. In 2012, Upson *et al*. (2012) published data to suggest that PR-B showed promise as a biomarker of progestin response. They performed a nested case–control study of women with CH and atypical hyperplasia who received treatment with oral progestins. Several biomarker candidates were investigated for protein expression and in women with atypical hyperplasia, the authors found higher PR-B expression in those with a 90% decreased risk of lesion persistence/progression (Upson *et al*., 2012).

#### Cyclooxygenase-2

Cyclooxygenase-2 (COX-2), also known as Prostaglandin-Endoperoxide Synthase 2 (PTGS2), is an isoform of the cyclooxygenase (COX) enzyme. It is involved in the conversion of arachidonic acid to prostaglandin H2, leading subsequently to the production of prostaglandin E2 (PGE2) (Erkanli *et al*., 2007; Faloppa *et al*., 2014). PGE2 has an established role in cell growth and development (Faloppa *et al*., 2014). In normal endometrial physiology, the expression of COX-2 and the metabolizing enzyme 15-Hydroxyprostaglandin Dehydrogenase (PGDH) are both regulated by progesterone (Baird *et al*., 1996; Hapangama *et al*., 2002). Increased COX-2 and PGE2 expression have been demonstrated to play key roles in the development of several malignancies (Williams *et al*., 1999) including EC (Tong *et al*., 2000; Jabbour and Boddy, 2003).

From our literature review, six studies investigated COX-2 expression within EH tissues (Supplementary Table SI). Three studies described trends of increasing expression of COX-2 from EH to EC (Orejuela *et al*., 2005; Erkanli *et al*., 2007; Nasir *et al*., 2007). Erkanli *et al*. (2007) demonstrated statistically significant COX-2 overexpression in EH and EC cases compared to PE. Orejuela *et al*., in their investigation of 43 retrospective endometrial biopsies, reported that COX-2 expression followed a trend of increased expression in EC and EH compared to normal endometrium. Their results, however, did not reach statistical significance and they recommended further studies utilizing much larger sample sizes. Nasir *et al*. performed qualitative and semi-quantitative COX-2 immunohistochemical staining scores based on the proportion of immunoreactive cells and the strength of cytoplasmic COX-2 expression (Nasir *et al*., 2007). These authors found increasing expression of COX-2 from EH to invasive ECs and concluded that COX-2 inhibition may have a potential utility to halt the progression of precursor lesions to invasive ECs (Nasir *et al*., 2007).

Faloppa *et al*. (2014) investigated COX-2 and nuclear factor-κB (NF-κB) expression in hyperplastic and malignant samples utilizing EIN diagnostic criteria, finding no significant difference in COX-2 expression between benign hyperplasia and EIN following *post hoc* analysis (Faloppa *et al*., 2014). Cao *et al*. (2002) demonstrated negative COX-2 expression in normal and hyperplastic endometrium.

Steinbakk *et al*. (2011b) published a research paper with associated review of potential EH biomarkers of progression to EC, in which they concluded that combining the morphometric *D*-score with negativity for COX-2 strongly predicted progression of EH to EC (Supplementary Table SII). To support this assertion, the authors described 8 out of 13 cases with a *D*-score < 1 (i.e. high progression risk for EC) and COX-2 negativity that progressed to EC as compared to 3 of 139 of all other cases (*P* < 0.0001) (Steinbakk, *et al*., 2011b).

Given the complex interplay between prostaglandins and steroid hormone responsiveness (reviewed in Wallace *et al*., 2010), it is maybe unsurprising that COX-2 is reported as a potential biomarker of progression of EH to EC. However, it is important to note that its role as a diagnostic biomarker for EH requires further investigation in well-characterized patient samples.

### Tumour suppressors

#### Phosphatase and tensin homologue

Phosphatase and tensin homologue (PTEN) is a tumour suppressor gene, located on chromosome 10q23, that encodes a dual-specificity phosphatase with both protein and lipid actions (Latta and Chapman, 2002). PTEN regulates cellular proliferation and apoptosis, acting as an antagonist to growth factor-induced intracellular signalling pathways (Kimura *et al*., 2004; Allison *et al*., 2008). Loss-of-function mutations of the *PTEN* gene can cause up-regulation of endometrial glandular proliferation *via* the PI3K/Akt/mTOR pathway and evidence for an association with EH and endometrioid EC has been demonstrated using heterozygous *Pten* knockout mice (Stambolic *et al*., 2000; Wang *et al*., 2002; Daikoku *et al*., 2008).

**Figure 6:**
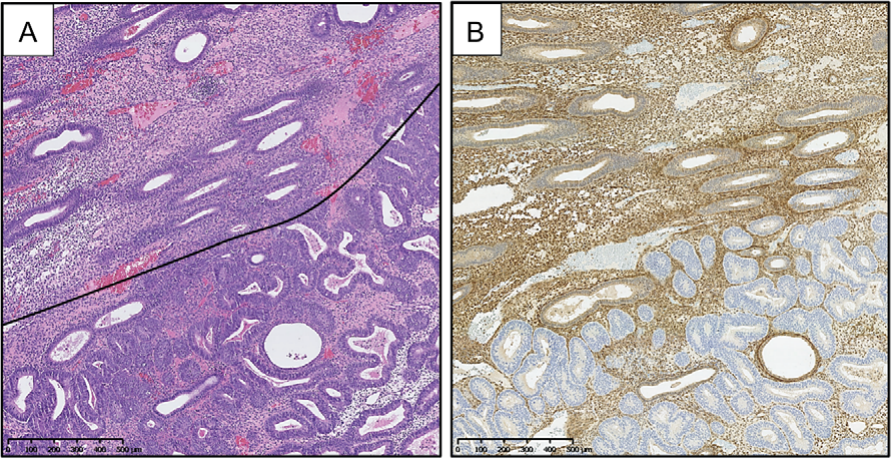
PTEN immunohistochemical staining of an EIN lesion. (**A**) H&E stained endometrial biopsy section demonstrating a region of EIN (below black line). (**B**) PTEN immunohistochemical staining of the same tissue section as in A. PTEN-null glands demonstrated by a loss of brown (DAB) cytoplasmic and nuclear staining in the same region corresponding to the EIN lesion as seen in image A. (Mouse monoclonal anti-human PTEN clone 6H2.1, Dako, Ely, UK; Antigen retrieval: decloaking chamber in citrate pH6; Overnight incubation 1:300 at 4°C.) Magnification: see scale bars.

PTEN protein has been evaluated across the normal menstrual cycle with changes in concentrations occurring in response to changes in the hormonal environment across the different phases (Mutter *et al*., 2000a). PTEN expression is increased in both the glandular epithelium and stromal compartments during the proliferative phase (plausibly influencing proliferation), whilst decreased in the glandular epithelial compartment during the secretory phase (Mutter *et al*., 2000a). Numerous studies have investigated PTEN expression in EH and EC using immunohistochemical techniques, with mixed reports regarding the pattern of expression seen (an example of loss of PTEN expression in EIN from our own work can be seen in Fig. 6).

Several authors have demonstrated that immunohistochemical loss of glandular PTEN expression is more marked in endometrioid EC and EIN compared to PE and benign EH (Mutter *et al*., 2000b; Baak *et al*., 2005b; Monte *et al*., 2010; Steinbakk *et al*., 2011b) (Supplementary Tables SI and SII). Mutter *et al*. (2000b) determined that PTEN mutations were evident in up to 55% of EIN lesions, suggesting that PTEN inactivation is an early event in EC carcinogenesis. Xiong *et al*. (2010) suggested that loss of PTEN expression is not a robust diagnostic marker of EIN, since they demonstrated complete PTEN loss occurring in only 38% of EIN lesions. Furthermore, Cirpan *et al*. (2006) demonstrated no significant difference in ‘complete loss’ of PTEN expression between PE, EIN and endometrioid EC, with some differences shown with ‘incomplete-loss’ of PTEN expression. These two studies raise the question as to what constitutes ‘complete loss’ of PTEN expression within a lesion, a discussion point considered by Allinson *et al*. in their 2008 review, highlighting that some authors regard PTEN-null as a single negative gland within a lesion whilst others consider only a more extensive loss.

Authors using WHO94 criteria have reported along similar lines to their counterparts using EIN criteria (Erkanli *et al*., 2006; Kapucuoglu *et al*., 2007; Sarmadi *et al*., 2009; Lee *et al*., 2012). Lee *et al*. found PTEN expression loss in endometrioid EC and CAH was higher than in SH. Kapucuoglu *et al*. echoed this finding; however, they noted no significant PTEN expression differences between CAH and EC, nor between individual EH groups (Kapucuoglu *et al*., 2007). One study, examining a small group of samples (*n* = 11), showed no significant difference between normal endometrium and EH (Kimura *et al*., 2004). However, this study analysed PTEN nuclear staining *via* a nuclear stain scoring system rather than reporting PTEN loss of expression (Kimura *et al*., 2004).

**Figure 7:**
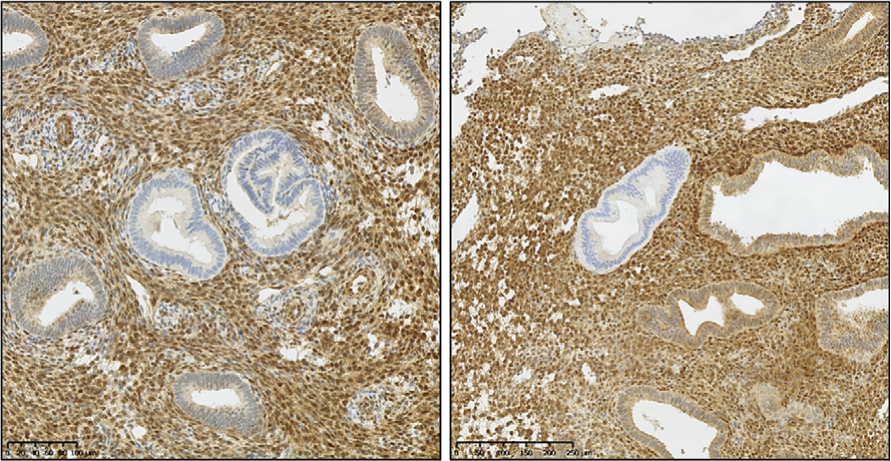
PTEN immunohistochemical staining of hyperplasia without atypia. PTEN immunohistochemical staining demonstrating isolated PTEN-null glands (loss of brown (DAB) staining) seen within two separate tissue sections diagnosed as hyperplasia without atypia. (Mouse monoclonal anti-human PTEN clone 6H2.1, Dako, Ely, UK; antigen retrieval: decloaking chamber in citrate pH6; overnight incubation 1:300 at 4°C.) Magnification: see scale bars.

Isolated PTEN-null glands have also been demonstrated within macroscopically normal premenopausal endometrial samples in a reported 43% of cases (Mutter *et al*., 2001) (an example of isolated PTEN-null glands can be seen in Fig. 7). These glands do not express PTEN protein owing to a genetic mutation and/or deletion and notably they persist between menstrual cycles (Mutter *et al*., 2001). That being said, the available evidence suggests only a small proportion of these macroscopically normal, PTEN-null glands will progress to endometrioid EC (Ayhan *et al*., 2015). Important evidence was gathered during a study that compared PTEN immunohistochemistry using samples from a cohort of women with EIN or EC, as well as histologically benign biopsies taken from the same women (matched non-neoplastic controls were included) (Mutter *et al*., 2014). Where PTEN-null glands were identified in both the index neoplastic biopsy and historic ‘normal’ biopsy, DNA sequencing was performed on both samples for comparative PTEN somatic mutation analysis. The results demonstrated that in only 6.7% of cases the PTEN-null, macroscopically normal glands were the direct progenitors of the high-risk neoplasia subsequently detected (Mutter *et al*., 2014).

Three studies identified by this review retrospectively analysed PTEN expression in women who subsequently progressed from EH to EC and two studies looked at women who had a concurrent/coexisting EC after a biopsy result of EH (Supplementary Table SII). Steinbakk *et al*. noted lower PTEN expression in EC samples than in EH samples and using a univariant analysis suggested that PTEN negativity in EH was prognostic of progression to EC (*P* = 0.026) (Steinbakk *et al*., 2011b). Lacey *et al*. (2008a) concluded that loss of PTEN expression in EH was neither sensitive nor specific in predicting progression to EC. Baak *et al*. (2005b) determined in their study that all EH cases that progressed to EC were PTEN-null; however, only 16% of all PTEN-null cases progressed to EC. They concluded that the prognostic power of PTEN could be increased when combined with tissue analysis using the morphometric *D*-score (Baak *et al*., 2005b). The two studies of women who had a concurrent/coexisting EC after a biopsy result of EH were more divisive. Pavlakis *et al*. (2010) noted that loss of PTEN expression on its own was not predictive of concurrent EC, however that changed when analysed in conjunction with a finding of marked nuclear atypia within an EIN lesion. Orbo *et al*. (2003) found loss of function of PTEN was more likely in EH lesions when a concurrent EC was present or when EC subsequently developed.

When considered in isolation, PTEN immunostaining continues to elicit conflicting views when it comes to its utility as a biomarker of EH and its value as a predictor of progression from EH to EC. Undoubtedly, its role in the process of endometrial carcinogenesis is still of great importance and so investigation into including its evaluation in any biomarker ‘profile’ should be encouraged.

#### Tumour protein p53

Tumour protein p53, or simply p53, is a protein encoded by the *TP53* gene that in humans is located on the short arm of chromosome 17 (17p13.1) (Isobe *et al*., 1986). When cellular DNA is damaged, the p53 protein regulates cell-cycle inhibition and apoptosis, thereby determining whether or not the damaged DNA should be repaired or destroyed (Ozkara and Corakci, 2004). Owing to its role in preventing damaged DNA from dividing, p53 is considered a tumour suppressor and is often referred to as ‘the guardian of the genome’ (Lane, 1992).

Loss of expression of wild-type p53 due to mutation or gene inactivation leads to malignant transformation of tissues (Cinel *et al*., 2002). In ECs, most *TP53* mutations are missense mutations, generally detected in serous/‘type 2’ ECs and associated with formation of a functionally defective p53 protein that is more stable and has a longer half-life than the wild-type p53 protein (Soong *et al*., 1996). The mutated protein product accumulates and is detected as overexpression in nuclei using immunohistochemistry. Typically wild-type p53 in cells cannot be detected by immunohistochemistry; however, if p53 is stabilized, due to overexpression in normal cells in response to DNA damage, a positive immunohistochemistry reaction (usually focal, weak and heterogeneous) can be detected in the absence of any mutation (Soong *et al*., 1996). To further complicate matters, nonsense or frame-shift mutations of *TP53* can lead to a protein undetectable by immunohistochemistry and so a completely negative p53 immunohistochemistry reaction may also indicate a gene mutation (Garg *et al*., 2010).

During the course of this review, we identified several studies that had assessed p53 immunohistochemical expression in EH and EC (Supplementary Tables SI and SII). Horrée *et al*. noted p53 expression gradually increasing from nearly all negative cells in inactive endometrium, through to EH where only a few cells were positive, with the highest expression seen in ECs (Horrée *et al*., 2007). Both Cinel *et al*. and Elhafey *et al*. demonstrated higher expression scores in atypical hyperplasias, with the highest expression scores in non-endometrioid EC (Elhafey *et al*., 2001; Cinel *et al*., 2002).

In terms of using p53 as a marker of progression from EH to EC, the only study our review captured was by Steinbakk *et al*. Their retrospective analysis demonstrated that two out of eight patients who developed EC from EH had ≤1% positivity for p53 which, using a univariant analysis, they found was prognostic of progression (*P* = 0.038). Although, given the small number of patients progressing to EC captured by this study, the CI is notably wide (0.9–23.2) (Steinbakk *et al*., 2011b).

Overall, given the known complexities associated with p53 immunohistochemical analysis, especially the misinterpretation of wild-type p53 staining, its use as a biomarker for EH diagnosis and progression to EC seems doubtful. This is in stark contrast with difficult to diagnose serous ECs or mixed ECs where a clear p53 overexpression pattern provides valuable diagnostic information.

#### AT-rich interactive domain-containing protein 1 A

AT-rich interactive domain-containing protein 1 A (ARID1A), also known BAF250A, is an important component of the SWItch/Sucrose Non-Fermentable (SWI/SNF) nucleosome remodelling complex. It is encoded by *ARID1A* which is located on chromosome 1p36.11 (Takeda *et al*., 2015). The SWI/SNF complex is involved in the regulation of cellular differentiation, tissue development and DNA repair (Guan *et al*., 2011; Werner *et al*., 2012). *ARID1A* is required for SWI/SNF complexes to suppress DNA synthesis and as such ARID1A is considered a tumour suppressor since it regulates cell proliferation and functions to prevent genomic instability (Mao and Shih, 2013). Mutations of *ARID1A* have been described in ~29–40% of cases of EC (Guan *et al*., 2011; Wiegand *et al*., 2011; Kandoth *et al*., 2013) *ARID1A* mutations are normally insertions or deletions that lead to the formation of truncated proteins (Guan *et al*., 2011).

Three studies identified by our review have investigated the role of ARID1A expression within EH tissues (Supplementary Table SI). Mao *et al*. (2013) performed an immunohistochemical investigation of 246 endometrial tissue samples spanning a range from normal endometrium to CAH and high-grade endometrioid EC. They specifically analysed tissues for ‘clonal’ loss of ARID1A, rather than loss of expression across the entire tissue section (Mao *et al*., 2013). The authors reported that all samples of normal endometrium retained ARID1A expression, with 16% of CAH demonstrating clonal but not complete loss of expression. In contrast, the samples lacking expression of ARID1A increased with EC tumour grade, from 25% in low-grade to 44% in high-grade tumours (Mao *et al*., 2013). The same group went on to compare ARID1A expression, along with that of PTEN and the proliferation marker Ki67, utilizing a cohort of 114 endometrial samples with a diagnosis of atypical hyperplasia/EIN (Ayhan *et al*., 2015). They noted that all specimens (*N* = 17) with focal ARID1A loss also exhibited concurrent loss of PTEN expression and that this was correlated with a significant increase in proliferation when compared to adjacent areas in the same tissue without concurrent loss of both markers (Ayhan *et al*., 2015). The authors used these findings to suggest that ARID1A may act to prevent PTEN inactivation from furthering cellular proliferation in the transition from pre-malignancy to EC (Ayhan *et al*., 2015).

Werner *et al*. adopted a semi-quantitative intensity staining score when analysing ARID1A expression in their retrospective study of 679 endometrial tissue samples (*n* = 641 ECs, *n* = 38 EH). Their findings echoed those of Mao *et al*. demonstrating a stepwise reduction in staining intensity of ARID1A with progression from hyperplasia without atypia (no loss of protein expression) to hyperplasia with atypia (16% loss of expression) and endometrioid tumours (19% loss of expression) (Werner *et al*., 2012).

ARID1A has emerged from molecular and genomic studies as an important candidate and tumour suppressor in gynaecological malignancies (Kandoth *et al*., 2013; Takeda *et al*., 2015). The current literature suggests we should recognize the potential of ARID1A as a valuable biomarker of progression from EH to EC and it, therefore, warrants further investigation in larger (more diverse) tissue sample sets.

### Transcription factors

#### Paired box gene 2

Paired box gene 2 (PAX2) is a member of a large gene family of pair box genes that are implicated in transcriptional regulation during the process of embryogenesis (Mansouri *et al*., 1994; Ryan *et al*., 1995). *PAX2* gene expression has been connected to the normal growth of the central nervous system, eyes, ears and urogenital system (Allison *et al*., 2012). Expression of PAX2 has been described as a marker of the Müllerian duct derivatives (fallopian tubes, uterus, cervix and upper vagina) (Tong *et al*., 2007). According to Tong *et al*. epithelial cells within the uterine glands normally demonstrate nuclear expression of PAX2 (Tong *et al*., 2007). The interest in PAX genes as a predictor of EH/EC have been stimulated by reports that they can act as proto-oncogenes through regulation of cell proliferation, survival and apoptosis (Robson *et al*., 2006; Lang *et al*., 2007).

**Figure 8:**
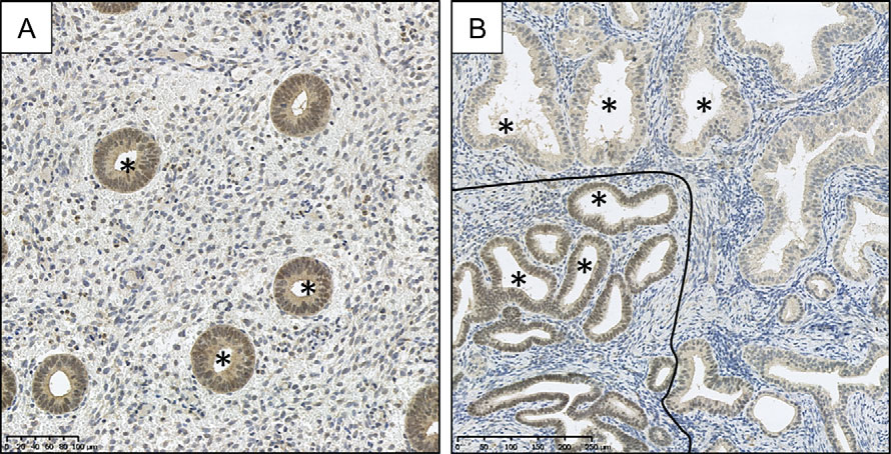
PAX2 immunohistochemical staining. Selection of glands marked by * in lumen. (**A**) PAX2 stained section of proliferative endometrium demonstrating strong brown nuclear (DAB) PAX2 staining within the glands, (**B**) loss of brown (DAB) PAX2 nuclear staining within the glands of an EIN lesion above/right of the black line. Crowded glandular background endometrium can also be seen. (Rabbit anti-PAX2 polyclonal Z-RX2, Invitrogen, Camarillo, CA; Antigen retrieval: decloaking chamber in citrate pH6; Overnight incubation 1:500 at 4°C.) Magnification: see scale bars.

Loss of PAX2 expression has been implicated in the development of EIN (Fig. 8) by several authors and has found potential utility as a tool when diagnosing difficult EIN cases (e.g. where there is no ‘normal’ tissue in a sample to act as in internal control when assessing nuclear morphology) (Quick *et al*., 2012). Five studies were captured by our review of the literature (Supplementary Table SI). For example, in a recent study, Joiner *et al*. (2015) built on earlier recommendations that PAX2 aids EIN diagnosis (Quick *et al*., 2012) and compared the WHO94 and EIN classification systems for EH using PAX2 immunohistochemistry. In their study, the authors considered complete loss of nuclear staining, or reduced nuclear staining as compared to background endometrium, to be indicative of reduced PAX2 expression (Joiner *et al*., 2015). Reduced PAX2 expression was noted in 92% of EIN cases and 88% of atypical EHs. Although the authors concluded that loss of PAX2 immunoexpression is a useful finding when deciding whether lesions are premalignant, they also advocated careful comparison to H&E sections when considering the findings (Joiner *et al*., 2015). Loss of PAX2 expression was also found in 71% of EIN cases by Monte *et al*. and in 74% of atypical EHs by Allison *et al*. (Monte *et al*., 2010; Allison *et al*., 2012). Allison *et al*. proposed that PAX2 loss occurs early in the process of endometrial carcinogenesis as they did not detect loss of expression in their proliferative or secretory endometrial samples. They added the caveat that the expression pattern does not discriminate between diagnostic categories of EH since its expression is ubiquitously lost amongst all EH groups (Allison *et al*., 2012). Monte *et al*. (2010) also corroborated this, adding that the greatest stepwise change in PAX2 expression occurs between normal and premalignant endometrium. Only one study reported alternative findings; Kahraman *et al*. (2012) demonstrated an increase in PAX2 expression with progression from premalignant states to EC.

The available literature regarding PAX2 as a diagnostic biomarker of EH appears to suggest promise due to its ability to discern normal cycling endometrium from EH and EC; however, as emphasized by Quick *et al*., up to a third of EIN lesions may not demonstrate loss of PAX2 and thus caution should be exercised with its interpretation (Quick *et al*., 2012). However based on these reports, the utility of PAX2 in a panel of diagnostic biomarkers warrants further investigation.

#### Heart and neural crest derivatives expressed transcript 2

Heart and neural crest derivatives expressed transcript 2 (HAND2) belongs to the basic helix-loop-helix (bHLH) family of transcription factors; it plays crucial roles during embryological cardiac morphogenesis (VanDusen *et al*., 2014) and knockout mice are infertile due to failure of implantation (Li *et al*., 2011). In mice, *Hand2* has been shown to be a PR-regulated gene and its expression in endometrial stromal cells inhibits epithelial cell proliferation *via* suppression of several fibroblast growth factors (Li *et al*., 2011).

When Jones *et al*. (2013) conducted a comprehensive epigenome-transcriptome-interactome analysis, they found HAND2 was at the centre of the most highly ranked differential hotspot in EC, leading them to propose that epigenetic deregulation of HAND2 was a crucial step in endometrial carcinogenesis. They reported that methylation of *HAND2* was increased in premalignant endometrial lesions when compared to normal endometrium and that this was associated with a reduction in HAND2 expression (Jones *et al*., 2013).

Buell-Gutbrod *et al*. (2015) also hypothesized that HAND2 plays a role in the development of EH and Type 1 endometrioid EC (Supplementary Table SI). In their immunohistochemical study, 56 archival hysterectomy specimens with a known pathological diagnosis of either disordered proliferative endometrium, SH or CH with or without atypia and EC, were investigated for expression of HAND2 (Buell-Gutbrod *et al*., 2015). The results demonstrated a statistically significant (*P* ≤ 0.001) reduction in the stromal expression of HAND2 between benign endometrium and both simple and CH with atypia and EC (Buell-Gutbrod *et al*., 2015). But there was no statistically significant difference between benign endometrium and SH without atypia or disordered proliferative endometrium (Buell-Gutbrod *et al*., 2015). The authors noted that the HAND2 antibody cannot distinguish between SH with atypia, CH with atypia and EC (Buell-Gutbrod *et al*., 2015).

These studies suggest that this progesterone-regulated transcription factor shows promise as a potential diagnostic biomarker of EH with an ability to differentiate neoplastic from endocrine driven EH. However, further work needs to be undertaken on larger and more diverse sample cohorts.

### Mismatch repair

The DNA mismatch repair (MMR) pathways repair damaged DNA resulting from insertions/deletions in microsatellites, replication errors from damaged DNA polymerases and events such as single base mismatches (Poulogiannis *et al*., 2010; Diaz-Padilla *et al*., 2013). Microsatellites refer to repetitive nucleotide sequences within DNA, typically with 1–5 nucleotide repeats that can be repeats of mononucleotide, dinucleotide or larger units (Karamurzin and Rutgers, 2009).

Microsatellite instability (MSI) ensues where there is expansion or reduction in the length of microsatellite tracts in malignant tissue (Diaz-Padilla *et al*., 2013). Inactivation of any of the MMR genes (including MLH1, MSH2, MSH6 and PMS2) can cause MSI (Eshleman and Markowitz, 1996; Poulogiannis *et al*., 2010). The National Cancer Institute (NCI) consensus or ‘Bethesda’ panel was established as a panel of microsatellite markers to be used to diagnose MSI. Although initially designed for colorectal cancer, the system has also been adopted for use in the endometrium (Boland *et al*., 1998). The panel comprises five microsatellite loci: two mononucleotide markers and three dinucleotide markers. MSI-high tumours are defined by their instability at two or more of the five loci (or >30% of loci if a larger panel of markers is used), whereas MSI-low tumours show instability at only one locus out of the five (or in 10–30% of loci in larger panels). Microsatellite stable (MSS) tumours are those without instability at any loci (or <10% of loci in larger panels) (Vilar and Gruber, 2010). MSI-high status has been demonstrated to be an indicator of poor prognosis in International Federation of Gynecology and Obstetrics (FIGO) stage 1, but not FIGO 2–4 endometrioid ECs (Steinbakk *et al*., 2011a).

Deficiencies in the MMR system are observed in 25–30% of somatic ECs and are commonly associated with endometrioid histology (Hecht and Mutter, 2006). ECs are observed both somatically and in association with germline mutations in MLH1, MSH2, MSH6 and PMS2 genes, as part of Lynch syndrome or hereditary non-polyposis colorectal cancer (HNPCC) syndrome (Hendriks *et al*., 2006). In sporadic EC, MMR deficiency is mainly caused by hypermethylation of the MLH1 promoter, silencing its expression, thus leading to MSI. This is responsible for the lack of immunohistochemically detectable MLH1 protein expression in the majority of sporadic ECs with MSI (Simpkins *et al*., 1999). Woo *et al*. (2014) demonstrated the utility of MMR immunohistochemistry in their 2014 study of MMR proteins in women with EC.

Studies involving immunohistochemical expression of MLH1, MSH2 and MSH6 were captured by our literature search (Supplementary Tables SI and SII). Our search did not identify any studies examining PMS2 expression in EH lesions. Berends *et al*. suggest that loss of MLH1 or MSH2 protein may be an early event in endometrial carcinogenesis. Their study of 62 cases was interesting in that they looked at patients with EC who had germline HNPCC/Lynch syndrome mutations, patients with HNPCC and no EC and patients with EC without HNPCC. In patients with EH, both with and without a germline *MLH1* mutation, loss of the corresponding protein was detected using immunohistochemistry (Berends *et al*., 2001). In six cases of EH and concurrent EC, loss of MLH1 or MSH2 proteins was seen in both areas within the tissue (Berends *et al*., 2001). Hamid *et al*. analysed endometrial samples from 123 women (including 51 EH cases) for MSH2 expression. They noted that all SHs showed a normal positive expression of MSH2, with some complex and atypical EHs demonstrating weak or no MSH2 expression at all (Hamid *et al*., 2002). However, this was not significant enough to be able to infer utility as a diagnostic marker for distinguishing between EH categories (Hamid *et al*., 2002). The authors also commented that loss of MSH2 expression is rarely observed in sporadic EC cases (Hamid *et al*., 2002). Orbo *et al*. looked at EC progression from EH and analysed expression of MLH1, MSH2 and MSH6. They found loss of expression in these markers was higher in EH cases where there was either a concurrent EC or subsequent progression to EC (Orbo *et al*., 2003).

Molecular evidence connecting an absence of expression of MLH1 with tumour-specific promoter hypermethylation in EH has previously been described, suggesting ECs with MSI may acquire this feature as precancers (Jovanovic *et al*., 1996). Esteller *et al*. share this view; they found that aberrant MLH1 methylation is almost exclusively restricted to atypical EHs (Esteller *et al*., 1999). In addition, the group noted that the atypical EHs methylated at MLH1 which demonstrate a MSI phenotype are usually those also associated with a concurrent EC that also have MSI and MLH1 methylation (Esteller *et al*., 1999). These reports and the trends observed from the above literature imply a role for the detection and categorization of deficiencies in the MMR system within EHs, with suggestions that deficiencies in the MMR system may be useful in predicting malignant progression.

### Cell adhesion and signalling

#### Beta-catenin

Beta-catenin (β-catenin) is a protein encoded by the *CTNNB1* gene (Norimatsu *et al*., 2007) that plays a critical role in cell–cell adhesion and is a constituent of the Wnt pathway (Morin, 1999). Canonical Wnt signalling through β-catenin is thought to have a significant role in regulating cell and tissue proliferation, differentiation and carcinogenesis (Villacorte *et al*., 2012). In normal cells, β-catenin is rapidly degraded by the proteasome and factors that impair this turnover lead to an excess of cytoplasmic protein (Scholten *et al*., 2003). This results in simultaneous translocation of β-catenin into the cell nucleus, where it may form transcriptionally active complexes with T-cell factor/lymphoid enhancer factors (Tcf/Lef), resulting in activation of downstream targets (Morin, 1999; Scholten *et al*., 2003).

Seven studies were captured by this review as having analysed the immunohistochemical expression of β-catenin with respect to EH and EC (Supplementary Tables SI and SII). Saegusa *et al*. (2001) and Liao *et al*. (2009) both noted more intense nuclear expression of β-catenin in atypical EHs and EC compared to non-atypical/benign hyperplasia and normal endometrium. Furthermore, Moreno-Bueno *et al*. (2003) suggested that nuclear accumulation of β-catenin is characteristic of endometrioid EC and may be an early event in the carcinogenesis process. The EIN classification system was utilized by Norimatsu *et al*. (2007), who demonstrated 26% of EIN cases had strong nuclear staining of β-catenin, in contrast to normal endometrium where no expression was detected. Xiong *et al*. (2010) noted abnormal β-catenin expression in 10% of benign hyperplasia/non-EIN, rising to 50% and 67% in EIN and EC, respectively; they infer that detection of β-catenin may be of use in distinguishing benign hyperplasia from EIN. In terms of progression of EH to EC, Ashihara *et al*. (2002) noted that 55% of EH with a concurrent EC had positive or intensively positive β-catenin nuclear staining. Steinbakk *et al*., in their retrospective study, found that 40% of their EHs that progressed to EC demonstrated nuclear staining (two of five) (Steinbakk, *et al*., 2011b).

Changes in nuclear β-catenin expression between normal, neoplastic and frankly malignant endometrial tissues provide insight into the activation of the beta-catenin/Wnt signal transduction pathway, highlighting potential for further mechanistic studies into the role played by Wnt-dependent target genes in the process of EH progression and carcinogenesis.

#### E-cadherin

E-cadherin is a trans-membrane epithelial cell adhesion protein containing a cytoplasmic domain that connects the actin cytoskeleton *via* a complex with its related cytoplasmic proteins: α-, β- and γ-catenins (Carico *et al*., 2010). Several *in vitro* studies have associated low expression of E-cadherin with progression of malignancy and abnormal expression of E-cadherin and β-catenin have been implicated in the invasive and metastatic ability of various epithelial tumours (Hashimoto *et al*., 1989; Frixen *et al*., 1991).

Three studies were identified by our literature search relating to E-cadherin and neoplastic endometrial lesions (Supplementary Table SI). Two studies reported decreased expression of E-cadherin in EC compared with atypical hyperplasias (Moreno-Bueno *et al*., 2003; Carico *et al*., 2010). Carico *et al*. (2010) analysed the entire spectrum of endometrial lesions from benign to malignant, noting a progressive reduction in glandular expression at each stage, from normal endometrium through to EC. Moreno-Bueno *et al*. (2003) noted that in EC, the largest reduction in E-cadherin expression is seen in high-grade malignancies. In contrast Ahmed *et al*. reported E-cadherin expression was higher in EC than in atypical hyperplasia (Ahmed and Muhammad, 2014).

These studies suggest a role for E-cadherin in regulating cell adhesion during endometrial carcinoma progression; however, only limited information can be drawn at present from the current literature on the utility of E-cadherin as a diagnostic marker of EH or for its capacity to predict progression to EC.

### Regulators of cell survival or migration

#### B-cell lymphoma 2/Bcl-2 associated x protein

Programmed cell death (apoptosis) plays an essential role in homeostatic mechanisms during cyclical endometrial breakdown, piecemeal shedding and tissue restoration at menses (Harada *et al*., 2004; Marshall *et al*., 2011). The B-cell lymphoma 2 (Bcl-2) gene forms part of a group of proto-oncogenes that extend cellular longevity by counteracting the apoptotic process (Reed, 1994). In contrast, the Bcl-2 associated x protein (BAX) gene is an apoptosis-promoting member of the Bcl-2 gene family (Oltvai *et al*., 1993). It is thought that the Bcl-2 and BAX proteins forms heterodimers *in vivo* (Kokawa *et al*., 2001), and the cellular Bcl-2: BAX ratio is an important factor in the regulation of apoptosis, with a high ratio resulting in cells becoming resistant to apoptotic stimuli and a low ratio inducing cell death (Sedlak *et al*., 1995; Vaskivuo *et al*., 2002). The BAX protein is expressed throughout the menstrual cycle, but Bcl-2 appears to be regulated by oestrogen, and demonstrates a rise in the proliferative phase, before falling to a plateau in the secretory and menstrual phases (Saegusa *et al*., 1996; Critchley *et al*., 1999; Allison *et al*., 2008).

Many authors have studied expression of Bcl-2 and BAX in the hyperplastic endometrium and EC (Supplementary Tables SI and SII). Four studies from our literature search demonstrated reduced Bcl-2 expression with a trend from normal endometrium (highest expression), through the hyperplasias, to EC (lowest expression) (Peiró *et al*., 2001; Risberg *et al*., 2002; Sakuragi *et al*., 2002; Vaskivuo *et al*., 2002). In addition, three studies noted Bcl-2 expression to be higher in non-atypical hyperplasias compared with atypical hyperplasias (Morsi *et al*., 2000; Kokawa *et al*., 2001; Nunobiki *et al*., 2009). Mitselou *et al*. (2003) referred to ‘adenomatous hyperplasia’ in their cohort, but also found lower levels of expression of Bcl-2 in the EC group. Cinel et al. (2002) published findings to the contrary noting a pattern of increasing expression between the EH groups. BAX immunohistochemistry expression was more divisive with two studies demonstrating an increasing trend from normal endometrium, *via* EH, to EC (Kokawa *et al*., 2001; Peiró *et al*., 2001) and two studies suggesting the inverse (Sakuragi *et al*., 2002; Vaskivuo *et al*., 2002). Kapucuoglu *et al*. (2007) found no significant change in BAX expression between EH groups, nor between EH and EC.

From the studies described above, it seems that increased Bcl-2 expression is a feature of benign EHs with a reduction in atypical EHs and ECs. Since Bcl-2 promotes cell survival, this pattern would fit with the observation of increased apoptosis in atypical EHs and ECs (Arends, 1999). In terms of utility as a EH biomarker, although unlikely to be of great diagnostic benefit on its own, the reports suggest that Bcl-2 expression may have some utility as a marker of EH progression.

### Others

Additional candidate markers deemed of potential relevance, which were identified by our review of the literature, are Survivin, p16, p21 and p27. The literature findings for these markers are summarized in Supplementary Tables SI and SII.

## Conclusions and what is still missing: towards a genomic future?

### Ongoing challenges

**Figure 9:**
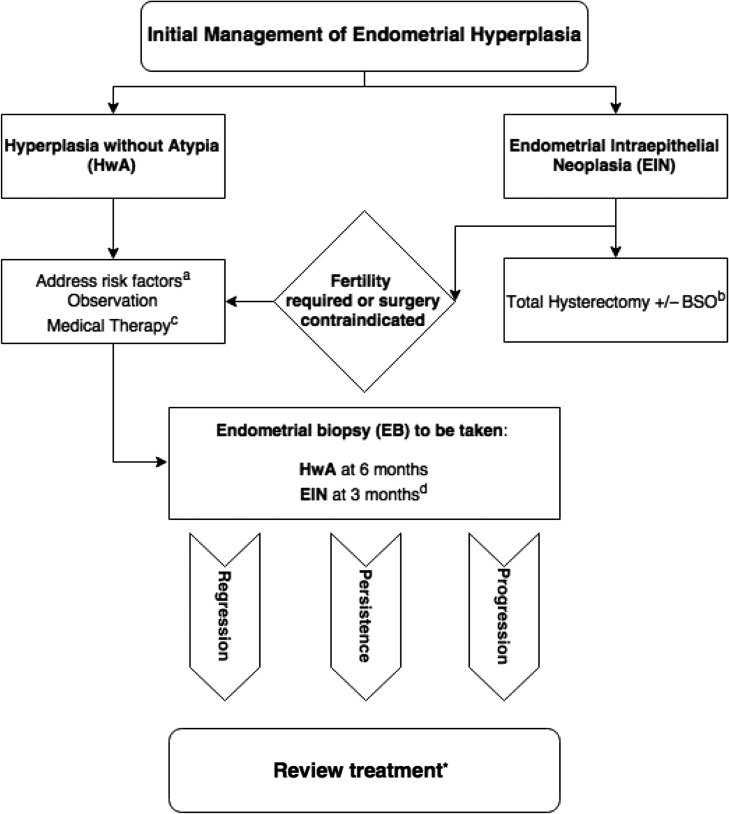
Flow diagram detailing proposed initial management of EHs based upon guidance released by the Royal College of Obstetricians and Gynaecologists (RCOG), UK in 2016 (Gallos *et al*., 2016). *A comprehensive treatment review and plan for ongoing management should be made at this point dependant on the outcome of the preceding endometrial biopsy and in keeping with local and national guidelines/practise. ^a^Risk factors for EH are specified in Table I. ^b^Ovarian conservation should be considered according to patients age, menopausal status and preferences. Total hysterectomy may also be indicated where there are (i) adverse effects with medical treatments, (ii) concerns over medication compliance and (iii) patient preference, e.g. elevated anxiety. ^c^Medical progestin therapy has varying forms; for further recommendations, refer to national guidelines (Committee on Gynecologic Practice, 2015; Gallos *et al*., 2016). ^d^Follow-up intervals for patients undergoing medical treatment of EIN should be tailored to individual patients and must reflect any ongoing risk factors, symptomatology and treatment responses. BSO = bilateral salpingo-oophorectomy.

A biopsy diagnosis of EIN carries a 45 times greater risk of progression to endometrioid EC after 1 year (Lacey *et al*., 2008b), meaning that surgery in the form of a total hysterectomy is usually the treatment of choice (Trimble *et al*., 2012). Alternatively, if a patient's co-morbid status or fertility wishes preclude hysterectomy, treatment with progestins and a programme of repeat observation can be considered, *albeit* with caution (Fig. 9).

It is highly likely that difficult EH/EIN clinical scenarios will become more commonplace in the future, due to escalating levels of obesity, the ageing population and increasing trends in delaying childbearing (Mackintosh and Crosbie, 2013; Daniluk and Koert, 2016). The clinical need for robust diagnostic biomarkers, capable of (i) differentiating neoplastic EIN lesions from benign hyperplasia and (ii) predicting their progression to EC, has, therefore, never been more apparent. For example, a premenopausal nulliparous patient with PCOS, found to have EIN on endometrial biopsy and who wishes to have future children, would benefit from a biomarker test able to predict the likelihood of her EIN progressing to EC *versus* EIN lesion involution. In addition, a diagnostic biomarker test may aid pathological analysis of sub-diagnostic EIN lesions, as described by Owings *et al*. (2014), whereby crowding of cytologically suspicious glands is a concern; however, the overall lesion size may be insufficient for EIN diagnosis. To date, a single ‘holy grail’ biomarker has not been found, although the search continues. Our review highlights several prominent immunohistochemical candidates from the current medical literature. We would hypothesize that moving forwards, a collective ‘panel’ approach of multiple candidates may provide greater diagnostic and prognostic value than what can currently be achieved by any single marker in isolation. This reflects the heterogeneous nature of these lesions, since the underlying pathogenesis is undoubtedly complex, multifactorial and may involve several different molecular pathways.

### Towards a genomic future?

Molecular classification is expanding our understanding of ECs and appears to correlate well with clinical outcome data, representing an important step forward from the traditional morphological diagnostic methods and the dichotomous ‘Type 1’ and ‘Type 2’ categories (Salvesen *et al*., 2009; Le Gallo *et al*., 2012; McConechy *et al*., 2012; Kandoth *et al*., 2013; Talhouk *et al*., 2015).

In 2013, an integrated molecular classification drawing on proteomic, genomic and transcriptomic analyses of over 370 ECs performed by The Cancer Genome Atlas (TCGA) resulted in new insights into EC subtypes (Kandoth *et al*., 2013). Briefly, employing array-based and sequencing methodologies, four major EC groups were characterized: (i) ultramutated cancers with DNA polymerase epsilon (*POLE*) mutations (7%), (ii) hypermutated cancers with MSI due to MLH1 promoter methylation (28%), (iii) ECs with low mutation rate and low frequency of DNA copy-number alterations (CNA, 39%) and (iv) ECs with low mutation rate but high-frequency DNA CNA (26%) (Kandoth *et al*., 2013). The TGCA suggested that EC patients harbouring *POLE* mutations (more commonly seen in Grade 3 endometrioid ECs in their cohort) had a less aggressive clinical course and improved progression-free survival when compared with the three other groups identified (Kandoth *et al*., 2013). In 2014, Meng *et al*. reported that *POLE* mutations could function as a prognostic marker for management of Grade 3 endometrioid EC. A suggested mechanism for these observations is that ECs with *POLE* mutations have an enhanced antitumor T-cell response combined with an enrichment of antigenic neopeptides (van Gool *et al*., 2015). Owing to the expense and technical expertise required when undertaking molecular classification of ECs, surrogate and clinically applicable methods for use with formalin-fixed paraffin-embedded tissues have been proposed (Talhouk *et al*., 2015). Through analysis of 152 historic ECs using a combination of p53 immunohistochemistry (as a surrogate marker for copy-number status), MMR (MLH1, MSH2, MSH6 and PMS2) immunohistochemistry and *POLE* mutation analysis, Talhouk *et al*. (2015) were able to replicate the survival curves as demonstrated by the TGCA.

Molecular classification has novel implications for diagnostics and personalizing treatment options for individual patients based upon prognostic outcomes. Given that the ECs seen in TGCA groups 1–3 were virtually all endometrioid (Kandoth *et al*., 2013), genomic profiling of EH/EIN as the precursor lesion to endometrioid EC may warrant further investigation. Owing in part to the escalating problem of obesity, rates of EHs and ECs and particularly the incidence of premenopausal disease are rising (Mackintosh and Crosbie, 2013; Wise *et al*., 2016). As already eluded to, these women potentially face difficult decisions regarding fertility limiting treatments and would, therefore, benefit from individualized risk stratification to help guide the decision-making process.

### Concluding remarks

**Figure 10:**
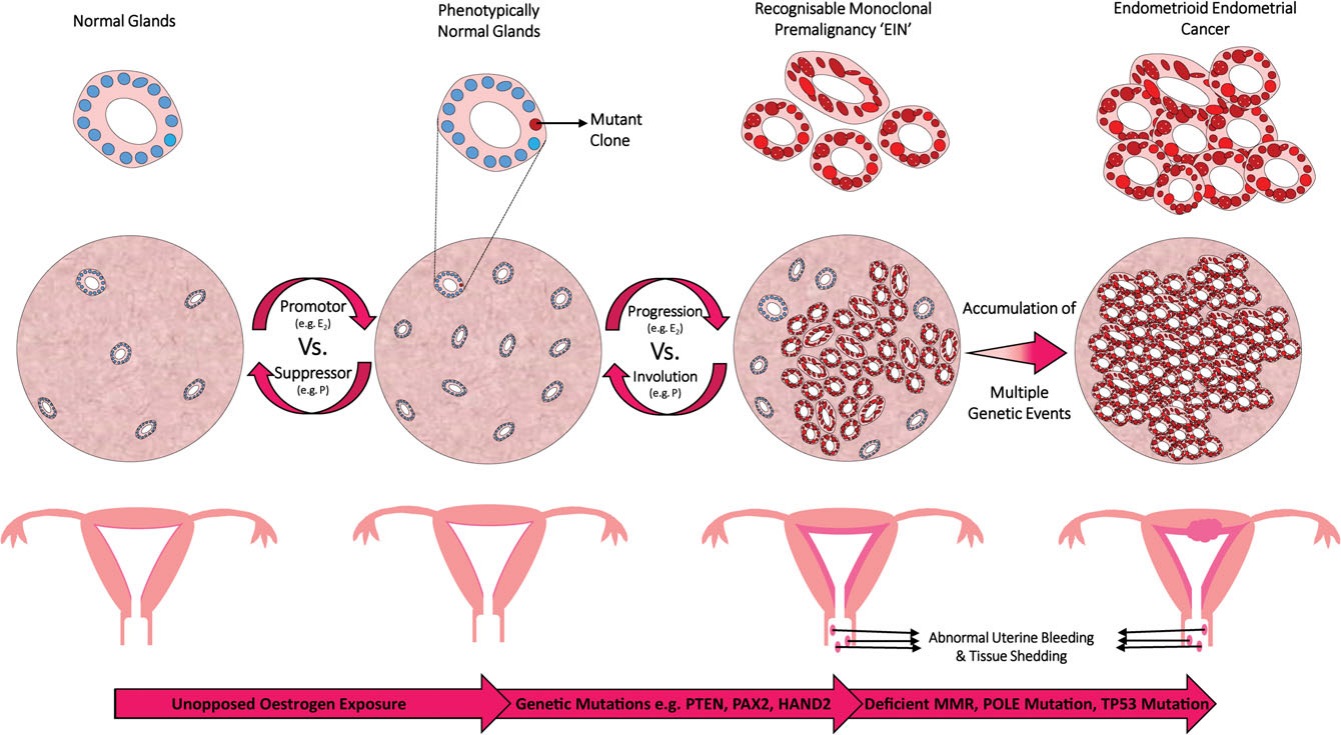
A schematic diagram to illustrate a proposed mechanism for monoclonal development of EIN. The endometrium is exposed to unopposed oestrogens *via* several possible routes (as described in Fig. 2). Oestrogen (E_2_), acting as a promotor, drives proliferation of the endometrial glands. This process can be reversible, e.g. with progestin (P) therapy acting as a suppressor. In ‘at risk’ individuals, a mutant clone may develop in this environment. The mutant clone occurs within phenotypically normal appearing endometrial glands. The mutant clone is selected for and progresses, aided by the influence of unopposed oestrogens. Over time, with the accrual of further genetic damage, not yet fully elucidated (bottom arrow shows suggestions), the mutant clone proliferates and an EIN lesion can be diagnosed during routine light microscopic examination of an H&E stained section. Endocrine modifiers can alter the balance of EIN progression *versus* involution. The patient may present with symptoms of abnormal uterine bleeding (AUB) and a thickened endometrium on ultrasound imaging. With the continued accumulation of further genetic damage, not yet fully elucidated (bottom arrow shows suggestions), the EIN lesion undergoes malignant transformation to EC.

EHs embody a uniquely heterogeneous group of lesions occurring in a dynamic, multicellular tissue that is exquisitely sensitive to changes in the hormonal environment. Historically, due to the heterogeneous nature of EH lesions, there has been considerable difficulty classifying them into clinically relevant and pathologically reproducible groups that correlate risk of malignancy with treatment options and clinical outcome. The introduction of EIN represents a fundamental change in our understanding of the development of EH (Fig. 10). EIN is considered a direct precursor lesion for the development of endometrioid EC, which is backed by molecular evidence that proposes a monoclonal lineage with significant malignant potential. The EIN system has been endorsed by the World Health Organisation in 2014 (Zaino *et al*., 2014).

We acknowledge that there may be a small number of candidate biomarkers that have fallen outside the search criteria employed by this review, despite every effort taken to include all relevant sources of information. This unfortunately is inevitable. Our focus on post-2000 literature aimed to ensure that we had the best coverage of current and up-to-date studies with an element of consistency between methodologies. We have chosen to focus on immunohistochemical biomarkers for this review. This technique is widely available and a there is a plethora of literature information detailing application of the technique in both EH and EC tissues. We felt that an attempt to summarize this in a comprehensive manner was needed. We recognize that there are limits to the conclusions that can be drawn when comparing a non-standardized semi-quantitative technique like immunohistochemistry, with researchers using different antibodies for the same antigen and employing different staining and scoring systems. In addition, the complexity of attempting to compare studies with a lack of consistency between diagnostic terminologies (e.g. EIN *versus* WHO94) has meant that a simple descriptive comparison is the ceiling of what can be achieved for the vast majority of cases.

The recent genomic classification of ECs heralds an exciting opportunity for EH risk stratification and, moving forwards, a role for qualitative genomic biomarkers is apparent. We are starting to see the age-specific incidence of EC increasing sharply at ages 40–44 years (Cancer Research UK, 2016) and what has previously been considered a predominantly postmenopausal disease is becoming an increasing concern for women of childbearing age, obese individuals and those with PCOS (Hardiman *et al*., 2003; Li *et al*., 2014; Shafiee *et al*., 2014). Since EH/EIN often precedes EC by several years (with approximate intervals dependent on the EH classification system adopted) the chance to intervene early with informative risk stratification and thus appropriate timely treatment is an opportunity not to be missed.

## Supplementary Material

Supplementary Data

Supplementary Data
