# Supplementary material for: New concepts for an old problem: the diagnosis of endometrial hyperplasia
Source: Hum Reprod Update. 2016 Dec 5;23(2):232–54. doi: 10.1093/humupd/dmw042 (PMC5850217; doi:10.1093/humupd/dmw042)
Supplement: Supplementary Data [file hru-16-0017-r1-supptable1.docx]

| Biomarker | Year | Cases (n) | Endometrial Hyperplasia Cases by Classification System | | | | | | | | | | | None Hyperplastic Tissue | | Antibody | Summary of Key Article Findings | Reference |
| --- | --- | --- | --- | --- | --- | --- | --- | --- | --- | --- | --- | --- | --- | --- | --- | --- | --- | --- |
|  |  |  | WHO 1994 / 2003 | | | | | | | | | EIN System | |  |  |  |  |  |
|  |  |  | DPEM | SH | | SAH | | CH | | CAH | | EH | EIN | EC | NE |  |  |  |
| PTEN | 2012 | 75 | - | 21 | | - | | - | | 22 | | - | - | 22 | 10 | PTEN (Clone 138G6), 1:100, Cell Signalling Tech | Authors found PTEN loss significantly higher in EC compared with SH. PTEN loss also higher in CAH compared with SH. | (Lee et al. 2012) |
|  | 2011 | 132 | 42 | 74 | | - | | - | | 16* | | - | - | - | - | PTEN, 1:800, Novocastra | Authors suggest PTEN may help determine progression risk in advanced EH. | (Pieczyńska et al. 2011) |
|  | 2010 | 305 | - | - | | - | | - | | - | | - | 52 | 62 | 191 | PTEN (Clone 6H2.1), 1:100, Dako | Authors found a non-significant PTEN loss in the sequence normal → premalignant → malignant. Greatest stepwise change is EIN → cancer transition. | (Monte et al. 2010) |
|  | 2010 | 117 | - | - | | - | | - | | - | | 59 | 24 | 24 | 10 | Unknown | Authors conclude a lack of PTEN expression not an appropriate marker for diagnosing EIN. | (Xiong et al. 2010) |
|  | 2009 | 87 | - | 21 | | - | | - | | 8 | | - | - | 29 | 29 | Anti-PTEN, 1:100, Zymed Labs | Authors conclude that PTEN expression significantly higher in cyclical endometrium than in atypical hyperplasia and EC. | (Sarmadi et al. 2009) |
|  | 2007 | 90 | - | - | | - | | - | | - | | 32 | 38 | - | 20 | PTEN (Clone 6H2.1), 1:100, Cascade Bioscience | Authors suggest that PTEN negative results may be reliable for detecting EIN when combined with positivity for beta-catenin. | (Norimatsu et al. 2007) |
|  | 2007 | 95 | - | 20 | | - | | 7 | | 10 | | - | - | 35 | 23 | Anti-PTEN (clone 17.A), 1:50, LabVision | Authors conclude that PTEN expression significantly higher in non-atypical hyperplasias than EC; no differences between CAH & EC or between individual hyperplasia groups. | (Kapucuoglu et al. 2007) |
|  | 2006 | 57 | - | - | | - | | - | | - | | 13 | 24 | 10 | 10 | Unknown | Authors demonstrate PTEN expression showed no differences among the cases of EIN, EC and PE | (Cirpan et al. 2006) |
|  | 2006 | 77 | - | 24 | | - | | 6 | | 8* | | - | - | 29 | 10 | PTEN (clone Ab-4, MS-21601-R7), Unknown dilution, Neomarkers | Authors show decreasing PTEN expression from PE → Hyperplasia → EC. | (Erkanli et al. 2006) |
|  | 2003 | 156 | - | 9 | | - | | 4 | | 7 | | - | - | 117 | 19 | Anti-PTEN (clone 28H6), 1:400, Novocastra | Authors use a PTEN staining score (intensity (0-4+) x percentage positive nuclei (0-4, in quartiles) & demonstrate no significant score differences between hyperplasia groups. | (Kimura et al. 2004) |
|  | 2000 | 72# | - | - | | - | | - | | - | | 7† | 12 | 33 | 20 | PTEN (Clone 6H2.1), 1:100, Cascade Bioscience | Authors suggest: 1) loss of PTEN is an early event in endometrial tumourigenesis 2) Individual PTEN-negative glands in oestrogen-exposed endometria are the earliest recognisable stage of endometrial carcinogenesis. | (Mutter et al. 2000) |
| PAX2 | 2015 | 67 | - | 7 | | - | | 25 | | 25 | | 15 | 50§ | - | - | PAX2, Unknown dilution, Cell Marque | Authors demonstrate loss of PAX2 correlated well with EIN. PAX2 loss useful adjunct when EIN/atypical hyperplasia in question. (NB/ Comparison between WHO and EIN classifications used). | (Joiner et al. 2015) |
|  |  |  | 10 Benign | | | | | | | | | 2 Benign | |  |  |  |  |  |
|  | 2012 | 52 | - | | - | | - | | - | | - | - | 52 | - | - | PAX-2 (Clone Z-R2X), 1:300, Invitrogen | Authors suggest PAX2 can be of use when delimiting EIN lesions. | (Quick et al. 2012) |
|  | 2012 | 121 | - | | - | | - | | 18 | | 19 | - | - | 47 | 37 (16) | PAX2 (pSer393), 1:100, Lifespan Biosciences | Authors note as the neoplastic lesion progresses from premalignant state to EC, PAX2 expression increases. (NB/ 16 Atrophic controls included). | (Kahraman et al. 2012) |
|  | 2012 | 203 | - | | 23 | | - | | 83 | | 54 | - | - | 15 | 28 | PAX-2 (Clone Z-R2X), 1:100, Zymed | Authors conclude PAX2 loss occurs early in the development of endometrial precancers & is not useful in distinguishing between diagnostic categories. | (Allison et al. 2012) |
|  | 2010 | 305 | - | | - | | - | | - | | - | - | 52 | 62 | 191 | PAX-2 (Clone Z-R2X), 1:300, Invitrogen | Authors found significant PAX2 loss in the sequence normal → premalignant → malignant. Greatest stepwise change is normal → premalignant transition. | (Monte et al. 2010) |
| HAND2 | 2015 | 56 | 1 | | 10 | | 2 | | - | | 10 | - | - | 22 | 11 | Anti-dHAND (sc-9409), 1:25, Santa Cruz | Authors found expression significantly reduced in atypical hyperplasias and EC. | (Buell-Gutbrod et al. 2015) |
| p53 | 2007 | 78 | - | | - | | - | | - | | - | 13 | 10 | 39 | 16 | p53, 1:400, Dako | Authors note expression gradually increases from normal (inactive) through hyperplasias to EC (NB/ Both EIN & WHO classifications used). | (Horrée et al. 2007) |
|  | 2004 | 92 | - | | 12 | | - | | - | | 12 | - | - | 38 | 30 | p53 (Clone DO-7+BP53-12;) 1 µg/ml, Neomarkers | Authors found no p53 expression in normal endometrium or hyperplasias. | (Ozkara & Corakci 2004) |
|  | 2002 | 56 | - | | - | | - | | 10 | | 3 | - | - | 29 | 14 | p53 (Clone D-07), Unknown dilution, Dako | Authors found no p53 expression in hyperplasias. 63% of endometrioid EC had focal or diffuse staining score. | (Sakuragi et al. 2002) |
|  | 2002 | 91 | 8 | | 9 | | 2 | | 4 | | 4 | - | - | 17 | 47 | p53 (Clone DO-7), 1:75, Dako | Authors show 52% of all hyperplasia & 65% EC positive for p53. Strongest staining seen in atypical hyperplasia and Grade 3 EEC. | (Cinel et al. 2002) |
|  | 2002 | 100 | - | | - | | - | | 30 | | 10* | - | - | 40 | 20 | p53 (PAb 1801), 1:50, BioGenex | Authors demonstrate no expression in non-atypical hyperplasias, 30% in atypical hyperplasia & highest in non-endometrioid cancers. | (Elhafey et al. 2001) |
|  | 2000 | 155 | - | | 10 | | - | | 16 | | 8* | - | - | 32 | 89 | p53 (Clone DO-7, IgG2b), 1:200, Ylem | Authors demonstrate no expression of p53 in hyperplasias. | (Ioachim et al. 2000) |
| β -Catenin | 2010 | 117 | - | | - | | - | | - | | - | 59 | 24 | 24 | 10 | Unknown | Authors demonstrate abnormal expression in 10% of benign hyperplasia, 50% of EIN and 67% of EC. Suggest β-catenin may be useful in distinguishing benign hyperplasia from EIN. | (Xiong et al. 2010) |
|  | 2009 | 146 | - | | 7 | | - | | 7 | | 37 | - | - | 80 | 15 | Anti-β-catenin (Clone 14), 1:1000, BD Transduction Lab | Authors found high nuclear staining in CAH and EC compared to hyperplasia without atypia and NE. | (Liao et al. 2009) |
|  | 2007 | 90 | - | | - | | - | | - | | - | 32 | 38 | - | 20 | β-catenin (clone17C2), 1:100, Novocastra | Authors found nuclear staining seen in 26.3% of EIN cases, with strong intensity. Not seen in NE. | (Norimatsu et al. 2007) |
|  | 2005 | 116 | - | | - | | - | | - | | 21 | - | - | 95 | - | Anti-β-catenin, 1:000, BD Transduction Lab | Authors suggest nuclear accumulation of β-catenin is characteristic of endometrioid EC & may be an early event as also present in atypical hyperplasia. | (Moreno-Bueno et al. 2003) |
|  | 2002 | 45 | - | | 10 | | - | | 7 | | 8* | - | - | 20 | - | Anti-β-catenin, 1:500, BD Transduction Lab | Authors demonstrate nuclear staining in 56% of hyperplasias & 55% of hyperplasias with concurrent EC. | (Ashihara et al. 2002) |
|  | 2001 | 411 | - | | - | | - | | 37^ | | 32* | - | - | 199 | 141 | Anti-β-catenin, 1:1000, BD Transduction Lab | Authors found nuclear expression higher in atypical hyperplasias and EC. | (Saegusa et al. 2001) |
| E-Cadherin | 2014 | 54 | - | | - | | - | | 5^ | | 14* | - | - | 28 | 7 | Anti-E-Cadherin (clone MS-1479R7), Dilution unknown, Thermo Scientific | Authors found significantly higher expression in EC than atypical hyperplasia. | (Ahmed & Muhammad 2014) |
|  | 2010 | 124 | - | | 24 | | - | | - | | 11* | - | - | 22 | 67 | E-Cadherin (HECD-1), dilution unknown, Zymed | Authors found decreasing expression of E-Cadherin from NE → SH→ Atypical Hyperplasia → EC. | (Carico et al. 2010) |
|  | 2005 | 116 | - | | - | | - | | - | | 21 | - | - | 95 | - | E-Cadherin (4A2C7), 1:200, Zymed | Authors demonstrate reduced expression in 40% atypical hyperplasias with highest reduction seen in none-endometrioid EC & high-grade endometrioid EC. | (Moreno-Bueno et al. 2003) |
| Bcl-2 | 2009 | 180 | - | | 30 | | 30 | | 30 | | 30 | - | - | 30 | 30 | Bcl-2 Oncoprotein, 1:400, Novocastra | Authors demonstrate that the expression level of Bcl-2 was significantly higher in hyperplasias without atypia when compared to hyperplasias with atypia. | (Nunobiki et al. 2009) |
|  | 2007 | 95 | - | | 20 | | - | | 7 | | 10 | - | - | 35 | 23 | Anti-Bcl-2 (Clone: 100/D5), 1/50, Neomarkers | Authors demonstrate a significantly higher expression of Bcl-2 in non-atypical hyperplasias compared with EC. No significant difference between atypical hyperplasias and EC noted. | (Kapucuoglu et al. 2007) |
|  | 2003 | 170 | - | | - | | - | | - | | - | - | - | 103 | 32 | Unknown | 35 Cases of ‘Adenomatous’ hyperplasia used in this study. Authors found expression of Bcl-2 lower in EC than the other groups. | (Mitselou et al. 2003) |
|  | 2002 | 111 | - | | 17 | | - | | 12 | | 23* | - | - | 28 | 31 | Anti-human Bcl-2, Unknown dilution, Dako | Authors describe a decreasing pattern of expression from NE → SH → CH → Atypical hyperplasia → EC. | (Vaskivuo et al. 2002) |
|  | 2002 | 56 | - | | - | | - | | 10 | | 3 | - | - | 29 | - | Bcl-2 (Clone 124), Unknown dilution, Dako | Authors describe decreasing expression of Bcl-2 from Proliferative Endometrium → Hyperplasia → EC. NB/ No expression seen in Secretory endometrium. | (Sakuragi et al. 2002) |
|  | 2002 | 91 | 8 | | 9 | | 2 | | 4 | | 4 | - | - | 17 | 47 | Bcl-2 (Clone 100/D5), 1:50, Neomarkers | Authors describe significantly higher Bcl-2 staining in hyperplasia compared to proliferative endometrium (PE), with no difference between hyperplasia groups noted. Staining also higher in EC than PE. | (Cinel et al. 2002) |
|  | 2002 | 194 | - | | 46 | | - | | 9 | | 5* | - | - | 41 | 54 | Anti-human Bcl-2, 1:100, Dako | Authors describe significantly lower Bcl-2 expression in EC than in hyperplasias. Highest expression seen in in proliferative endometrium. | (Risberg et al. 2002) |
|  | 2001 | 56 | - | | 10 | | 22 | | - | | - | - | - | 56 | 24 | Bcl-2 (Clone 124), 1:20, Dako | Authors examined tissue adjacent to n=56 concurrent EC. None significant reduction in Bcl-2 expression seen from atrophic endometrium to hyperplasia to EC. | (Peiró et al. 2001) |
|  | 2001 | 40 | - | | - | | - | | 9^ | | 7* | - | - | 20 | 4 | Anti-human Bcl-2 (clone 124, isotype IgG1), Unknown dilution, Dako | Authors describe stronger Bcl-2 expression in hyperplasia samples compared with EC and postmenopausal control. Significantly stronger expression seen in non-atypical compared to atypical hyperplasias. | (Kokawa et al. 2001) |
|  | 2000 | 107 | 6 | | 12 | | - | | - | | 8* | - | - | 29 | 52 | Bcl-2 (Clone 124), 1:50, Dako | Authors describe increased expression in hyperplasia tissues, becoming difficult to detect when atypia present and through to EC. | (Morsi et al. 2000) |
| BAX | 2007 | 95 | - | | 20 | | - | | 7 | | 10 | - | - | 35 | 23 | Anti-Bax (clone: 2D), 1/50, Neomarkers | Authors found no statistically significant differences in BAX expression between hyperplasias and hyperplasias and carcinomas. All normal endometrial samples expressed BAX. | (Kapucuoglu et al. 2007) |
|  | 2002 | 111 | - | | 17 | | - | | 12 | | 23* | - | - | 28 | 31 | Anti-human Bax, Unknown dilution, Pharmingen | Authors describe highest level of BAX expression seen in SH with decreasing levels seen thereafter from CH → atypical hyperplasia → EC. | (Vaskivuo et al. 2002) |
|  | 2002 | 56 | - | | - | | - | | 10 | | 3 | - | - | 29 | - | Bax (P19), 1:200, Santa Cruz | Authors described reduced BAX expression in EC irrespective of tumour grade. All hyperplastic samples expressed BAX. | (Sakuragi et al. 2002) |
|  | 2001 | 56 | - | | 10 | | 22 | | - | | - | - | - | 56 | 24 | Bax (Clone P(r)), 1:20, Oncogen | Authors describe a significant trend towards increased BAX expression from atrophic endometrium through the hyperplasias to EC. | (Peiró et al. 2001) |
|  | 2001 | 40 | - | | - | | - | | 9^ | | 7* | - | - | 20 | 4 | Anti-human Bax, Unknown dilution, Calibrochem | The authors report BAX expression was more frequent and stronger in cases of EC than that in postmenopausal endometrium and hyperplasia without atypia. | (Kokawa et al. 2001) |
| COX-2 | 2014 | 272 | - | | - | | - | | - | | - | 37 | 17 | 218 | - | Anti-COX-2, Clone NCL-COX-2, 1:1000, Novocastra | Post hoc analysis found no significant difference between EH and EIN expression of COX-2. Overall COX-2 expression lower in EC compared to non-malignant lesions. | (Faloppa et al. 2014) |
|  | 2007 | 36 | - | | - | | - | | 9^ | | 5 | - | - | 22 | - | Anti-COX-2, 1:100, Caymen Chemical | Authors suggest a strong expression of COX-2 in EC, & trend toward an increasing expression of COX-2 from EH to invasive ECs. | (Nasir et al. 2007) |
|  | 2007 | 100 | - | | 30 | | - | | - | | - | - | - | 50 | 20 | COX-2 (Clone SP21), Unknown dilution, Neomarkers | Authors report COX-2 expression was significantly increased in SH and EC compared to proliferative endometrium. | (Erkanli et al. 2007) |
|  | 2005 | 43 | - | | 10 | | - | | 4 | | 5 | - | - | 14 | 10 | COX-2, 1:50, Immunobiological | Authors suggest a possible trend toward increased COX-2 expression in EC and hyperplasia compared to NE – results not significantly different. | (Orejuela et al. 2005) |
|  | 2002 | 40 | - | | - | | - | | 6 | | - | - | - | 24 | 10 | COX-2 (PGHS-2), 1:400, Oxford | Authors report COX-2 expression not detected in benign and hyperplastic endometrium with high expression and intense staining in poorly differentiated EC. | (Cao et al. 2002) |
| p27 | 2013 | 75 | - | | 25 | | - | | - | | - | - | - | 25 | 25 | p27, Unknown dilution, Neomarkers | Authors report a significant progressive decrease in p27 expression from NE → SH → EC. | (Gezginc et al. 2013) |
|  | 2007 | 78 | - | | 7 | | - | | 9 | | 7 | 13 | 10 | 39 | 16 | p27, 1:500, Transduction | Authors report a non-significant decrease in expression from EIN to EC (NB/ Both EIN & WHO classifications used). | (Horrée et al. 2007) |
|  | 2006 | 77 | - | | 24 | | - | | 6 | | 8* | - | - | 29 | 10 | p27 (clone Ab-1), Unknown Dilution, Neomarkers | Authors demonstrate a significant decrease in expression between hyperplasia & EC cases compared with NE. | (Erkanli et al. 2006) |
|  | 2004 | 92 | - | | 12 | | - | | - | | 12 | - | - | 38 | 30 | p27 (Clone DCS-72.F6), 2 µg/ml, Neomarkers | Authors report expression in SH, high expression and CAH with significantly reduced expression in EC. | (Ozkara & Corakci 2004) |
|  | 2003 | 276 | - | | - | | - | | 24 | | 5^ | - | - | 217 | 30 | p27 (Clone Kip1), 1:50, Santa Cruz | Authors demonstrate significant loss of p27 expression from normal through hyperplastic endometrium to endometrial adenocarcinomas. | (Masciullo et al. 2003) |
| p21 | 2008 | 120 | - | | - | | - | | 20^ | | - | - | - | 20 | 60 | p21 (sc-6246), Unknown dilution, Santa Cruz | Authors report expression of p21 was undetectable in polyps and slightly increased to a low level in hyperplasia and carcinoma. (NB/ 20 Endometrial polyps include in cohort analysed). | (Cobellis et al. 2008) |
|  | 2007 | 78 | - | | 7 | | - | | 9 | | 7 | 13 | 10 | 39 | 16 | p21, 1:25, Dako | Authors demonstrate significant difference in expression between non-EIN hyperplasia and EIN with higher expression seen in EIN. (NB/ Both EIN & WHO classifications used). | (Horrée et al. 2007) |
|  | 2002 | 40 | - | | - | | - | | 6 | | - | - | - | 24 | 10 | p21 (Clone SX118), 1:50, Dako | Authors report overexpression of p21 in hyperplasia and EC with no overall trend. Lower expression seen in benign endometrium. | (Cao et al. 2002) |
| MLH1 | 2001 | 62 | - | | 2 | | - | | 5 | | 11 | - | - | 48 | - | MLH1 (G168-728), 1:500 PharMingen | Authors assessed n=10 HNPCC patients with EC, n=15 HNPCC patients without EC and n=38 EC patients without HNPCC. Study also shows that in endometrial hyperplasia from MLH1 mutation carriers with or without concurrent EC, loss of the corresponding protein can be observed. Also, in patients with endometrial hyperplasia and no proven Germline mutation, loss of MLH1 was found. In 6 patients with endometrial hyperplasia and concurrent EC loss of protein was seen in both tissues. Authors suggest that loss of MLH1 protein expression may be a common early event in endometrial carcinogenesis. | (Berends et al. 2001) |
|  |  |  |  |  | From EC with concurrent hyperplasia | | | | | | |  |  |  |  |  |  |  |
| MSH2 | 2002 | 123 | - | | 19 | | - | | 12 | | 20* | - | - | 27 | 45 | hMSH2 (Ab-2), 1:50, Oncogene Science | Authors found all SH showed positive immunoreactivity for MSH2. Authors note that loss of MSH2 expression is observed rarely in patients with sporadic EC. | (Hamid et al. 2002) |
|  | 2001 | 62 | - | | 2 | | - | | 5 | | 11 | - | - | 48 | - | MSH2 (Ab-2 ), 1:100, Calbiochem | Authors assessed n=10 HNPCC patients with EC, n=15 HNPCC patients without EC and n=38 EC patients without HNPCC. Study also shows that in endometrial hyperplasia from MSH2 mutation carriers with or without concurrent EC, loss of the corresponding protein can be observed. Also, in patients with endometrial hyperplasia and no proven Germline mutation, loss of MSH2 was found. In 6 patients with endometrial hyperplasia and concurrent EC loss of protein was seen in both tissues. Authors suggest that loss of MSH2 protein expression may be a common early event in endometrial carcinogenesis. | (Berends et al. 2001) |
|  |  |  |  |  | From EC with concurrent hyperplasia | | | | | | |  |  |  |  |  |  |  |
| ERα | 2011 | 131 | 41 | | 58 | | - | | 17 | | 15* | - | - | - | - | ERα, 1:200, Dako | Authors demonstrate a non-significant increase in ERα expression from DPEM to EH and decreased in AEH. The highest levels observed were in CH. | (Pieczyńska et al. 2011) |
|  | 2008 | 114 | - | | 30 | | - | | 13 | | 20* | - | - | 37 | 15 | Anti-ERα, 1:100, Santa Cruz Biotech | Authors demonstrate significant increase in ERα from Prolif → SH → CH. In atypia hyperplasia and EC, ERα was decreased significantly. Most EC expressed ERα, either alone or in combination with ERβ, and the ERβ / ERα ratio was decreased when compared to Prolif endometrium. | (Hu et al. 2008) |
|  | 2008 | 83 | - | | 20 | | - | | - | | - | - | - | 26 | 37 | ERα (Clone ID5), 1:100, Neomarkers | No significant differences seen in expression between SH and proliferative endometrium. | (Chakravarty et al. 2008) |
|  | 2005 | 92 | - | | 13 | | - | | 13 | | 12* | - | - | 32 | 22 | ERα (NCL-Er-6FF), 1:30, Novocastra | Authors describe significant difference between EC and each EH group. No significant difference in relation to ERα staining among hyperplastic groups. | (Bircan et al. 2005) |
|  | 2003 | 210 | - | | 30 | | 30 | | 30 | | 30 | - | - | 30 | 60 | ERα, 1:100, Dako | Authors describe a stepwise decrease in ERα expression from CH → CAH → EC, compared to NE. | (Nunobiki et al. 2003) |
|  | 2003 | 114 | - | | 12 | | - | | 12 | | 12* | - | - | 58 | 20 | Anti-ER (specific for ERα), 1:100,  Immunotec | Authors describe a significant reduction in ER expression in hyperplasia compared to proliferative endometrium. ER expression decreased with advancing EC grade. | (Uchikawa et al. 2003) |
| ERβ | 2008 | 83 | - | | 20 | | - | | - | | - | - | - | 26 | 37 | ERβ (Clone 14CA), 1:25, Gene Tex | Ni significant differences seen in expression between SH and proliferative endometrium. | (Chakravarty et al. 2008) |
|  | 2008 | 114 | - | | 30 | | - | | 13 | | 20* | - | - | 37 | 15 | Anti- ERβ. 1:100, Santa Cruz Biotech | Authors demonstrate significant increase in ERα from Prolif → SH → CH. In atypia hyperplasia and EC, ERα was decreased significantly. Most EC expressed ERα, either alone or in combination with ERβ, and the ERβ / ERα ratio was decreased when compared to Prolif endometrium. | (Hu et al. 2008) |
| PR | 2011 | 132 | 39 | | 56 | | - | | 19 | | 18* | - | - | - | - | PR, 1:200, Dako | Authors demonstrate PR expression the majority of analysed cases (minus one case of atypical EH) with intensity of staining reducing in hyperplasias. | (Pieczyńska et al. 2011) |
|  | 2004 | 70 | - | | - | | - | | 10^ | | 10* | - | - | 30 | 20 | PgR 636, 1:50, Dako | Authors demonstrate PR expression increases progressively from non-atypical hyperplasia to atypical endometrial hyperplasia, with PR expression in EC varying according to tumour grade. | (Ghabreau et al. 2004) |
|  | 2005 | 43 | - | | 10 | | - | | 4 | | 5 | - | - | 14 | 10 | PR, Unknown dilution, Novocastra | Authors demonstrate no significant difference in PR expression between normal endometrium and hyperplasias. Slight reduction seen from these groups compared to EC but none significant. | (Orejuela et al. 2005) |
|  | 2003 | 114 | - | | 12 | | - | | 12 | | 12* | - | - | 58 | 20 | Anti-PR, 1:100,  Immunotec | Authors describe a significant reduction in PR expression in hyperplasia compared to proliferative endometrium. PR expression decreased with advancing EC grade. | (Uchikawa et al. 2003) |
|  | 2003 | 210 | - | | 30 | | 30 | | 30 | | 30 | - | - | 30 | 60 | PgR, 1:50, Dako | Authors describe a stepwise decrease in PR expression from SH to G1 EC. | (Nunobiki et al. 2003) |
| Survivin | 2009 | 23 | - | | 4 | | 1 | | 10 | | 8 | - | - | - | - | Anti-survivin, 1:100, Dako | Authors analysed n=23 patients with endometrial hyperplasia pre & post progestin treatment. Survivin expression pre treatment demonstrated high expression across all hyperplasias. Survivin expression significantly decreased post progestin treatment in those that responded to the treatment only. | (Chen et al. 2009) |
|  | 2007 | 100 | - | | 30 | | - | | - | | - | - | - | 50 | 20 | Survivin (Clone Ab-5), Unknown dilution, Neomarkers | Authors report Survivin overexpression in SH and EC compared to proliferative endometrium | (Erkanli et al. 2007) |
|  | 2006 | 77 | - | | 24 | | - | | 6 | | 8* | - | - | 29 | 10 | Survivin (Clone Ab-2), Unknown dilution, Neomarkers | Authors show increasing Survivin expression from PE → Hyperplasia → EC | (Erkanli et al. 2006) |
| p16 | 2007 | 78 | - | | 7 | | - | | 9 | | 7 | 13 | 10 | 39 | 16 | p16, 1:160, Neomarkers | Authors demonstrate significant difference in expression between non-EIN hyperplasia and EIN with higher expression seen in EIN. (NB/ Both EIN & WHO classifications used). | (Horrée et al. 2007) |
| ARID1A | 2013 | 246 | - | | - | | - | | - | | 38 | - | - | 88L  55H | 51 | ARID1A (HPA005456), 1:250, Sigma-Aldrich | NB/ Also includes endometrial polyps, n=14. L= Low Grade, H= High Grade.  % complete ARID1A loss increased from 0% in CAH, to 25% in L-EC to 44% H-EC. Clonal loss also noted; 16% CAH, 24% L-EC. | (Mao et al. 2013) |
|  | 2013 | 650 | - | | - | | - | | 7^ | | 31 | - | - | 641  77M | - | ARID1A (AT1188a), 1:100, Biosite | NB/ EC’s included 77 metastatic lesions. Loss in hyperplasia without atypia 0%, CAH 16%, 19% primary ECs and 28% metastatic EC. | (Werner et al. 2012) |
|  | 2015 | 114 | - | | - | | - | | - | | - | - | 114 | - | - | ARID1A (HPA005456), 1:1000, Sigma-Aldrich | 70% PTEN expression loss, 15% focal ARID1A loss. All specimens with ARID1A loss had concurrent PTEN loss and this conferred a significant increased in Ki67 proliferation expression. | (Ayhan et al. 2015) |

*Atypical hyperplasia – Unclear if simple or complex

^Combined simple and complex hyperplasia without atypia / Non-atypical hyperplasia

†Unopposed oestrogen effect – Benign

#Immunohistochemistry cases only described here

§ Includes 11 cases of Focal Glandular Crowding (FGC) – i.e. lesions not large enough to be classified as EIN but still of significant risk

HNPCC = Hereditary Non-Polyposis Colorectal Cancer / Lynch Syndrome, DPEM = Disordered Proliferative Endometrium, SH = Simple hyperplasia without atypia, SAH = Simple atypical hyperplasia, CH = Complex hyperplasia without atypia, CAH = Complex atypical hyperplasia, EH = Benign hyperplasia / Non-EIN, EIN = Endometrial Intraepithelial Neoplasia, EC = Endometrial Cancer (Grades & types where required discussed in findings), NE = Normal cycling endometrium, PE = Proliferative Endometrium, WHO = World Health Organisation.
