# Supplementary material for: New concepts for an old problem: the diagnosis of endometrial hyperplasia
Source: Hum Reprod Update. 2016 Dec 5;23(2):232–54. doi: 10.1093/humupd/dmw042 (PMC5850217; doi:10.1093/humupd/dmw042)
Supplement: Supplementary Data [file hru-16-0017-r1-supptable2.docx]

| Biomarker | Year | Total EH Cases (n) | EH Classification System Used (WHO*/EIN) | EH Cases progressed to EC | Interval prior to EC diagnosis | Antibody | Summary of Article Findings | Reference: |
| --- | --- | --- | --- | --- | --- | --- | --- | --- |
| PTEN | 2011 | 152 | Both | 11 | 57 Months (Median) | PTEN (Clone 6h2.1), 1:300, Dako | Authors found lower PTEN expression in EC than EH. PTEN negativity deemed prognostic of progression to EC. | (Steinbakk et al. 2011) |
|  | 2010 | 83 | Both | 33† | 12 Weeks | PTEN (Clone 6h2.1), 1:300, Cascade Bio | Authors found that loss of PTEN expression, when combined with nuclear atypia within an EIN lesion, was predictive of concurrent EC in subsequent hysterectomy specimen. PTEN loss alone not predictive. | (Pavlakis et al. 2010) |
|  | 2008 | 379 | WHO | 138 | At least 1 Year (Median 6 Years) | PTEN (Clone 6h2.1). 1:300, Cascade Bio | Authors compared 138 EH cases that progressed to EC with 241 matched EH controls that did not progress. They found that loss of PTEN on endometrial biopsy was not a sensitive or specific marker of subsequent progression to EC. | (Lacey et al. 2008) |
|  | 2005 | 103 | EIN | 7 | 50 Months (Mean) | PTEN (Clone 6h2.1), 1:300, Cascade Bio | Authors found all EH cases progressing to EC to be PTEN negative, however of the total PTEN negative cases only 16% progressed to EC. Authors conclude that prognostic power of PTEN increased when combined with EIN criteria and more so with morphometric D-score. | (Baak et al. 2005) |
|  | 2003 | 68 | WHO | 18§ | 6.6 Months (Mean) | PTEN (Clone A2B1:Sc 7974), 1:50, Santa-Cruz | Authors found loss of function of PTEN higher in EH with co-existing EC / subsequent development of EC (NB/ Cohort with no progression to EC, follow-up interval 10-21 years). | (Orbo et al. 2003) |
| p53 | 2011 | 152 | Both | 11 | 57 Months (Median) | p53 (Clone, D07), 1:200, Dako | Authors found low expression of p53 in EH; rising from EH to EC. p53 <1% deemed prognostic of progression to EC. | (Steinbakk et al. 2011) |
| β -Catenin | 2011 | 152 | Both | 11 | 57 Months (Median) | β-Catenin (Clone 17C2), 1:300 Novocastra | Authors found nuclear staining of Beta-Catenin prognostic of progression to EC when compared to cytoplasmic/membrane staining. | (Steinbakk et al. 2011) |
| Bcl-2 | 2011 | 152 | Both | 11 | 57 Months (Median) | Bcl-2 (Clone Bcl-2/100/D5), 1:40, Novocastra | Authors found reduced Bcl-2 expression of < 80% in EH was prognostic of progression to EC. | (Steinbakk et al. 2011) |
| COX-2 | 2011 | 152 | Both | 11 | 57 Months (Median) | COX-2 (Clone 4H12), 1:400, Novocastra | Authors found negativity for COX-2 in EH was prognostic of progression to EC. Overall low expression in EH. | (Steinbakk et al. 2011) |
| p27 | 2011 | 152 | Both | 11 | 57 Months (Median) | p27 (Clone SX53G8), 1:100, Dako | Authors found low expression in EH, falling in EC and demonstrate expression of < 17% in EH was prognostic of progression to EC. | (Steinbakk et al. 2011) |
| p21 | 2011 | 152 | Both | 11 | 57 Months (Median) | p21 (Clone 4D10), 1:25, Novocastra | Authors found low expression in EH, rising between EH & EC. They demonstrate p21 expression not a prognostic in progression of EH to EC. | (Steinbakk et al. 2011) |
| MLH1 | 2003 | 68 | WHO | 18§ | 6.6 Months (Mean) | HMLH1 (Clone G168–15), 1:100 Transduction Lab | Authors found loss of expression in MLH1 higher in EH with coexisting EC / subsequent development of EC (NB/ Cohort with no progression to EC, follow-up interval 10-21 years). | (Orbo et al. 2003) |
| MSH2 | 2003 | 68 | WHO | 18§ | 6.6 Months (Mean) | HMSH2 (Clone G219–1129), 1:50 Transduction Lab | Authors found loss of expression in MSH2 higher in EH with coexisting EC / subsequent development of EC (NB/ Cohort with no progression to EC, follow-up interval 10-21 years). | (Orbo et al. 2003) |
| MSH6 | 2003 | 68 | WHO | 18§ | 6.6 Months (Mean) | HMSH6/GT BT (Clone 44), 1:400 Transduction Lab | Authors found loss of expression in MSH6 higher in EH with coexisting EC / subsequent development of EC (NB/ Cohort with no progression to EC, follow-up interval 10-21 years). | (Orbo et al. 2003) |
| Survivin | 2011 | 152 | Both | 11 | 57 Months (Median) | Survivin (Clone D8), 1:75, Santa Cruz | Authors found low expression in EH, rising between EH &EC. They demonstrate <21 Survivin positive cells prognostic in progression of EH to EC. | (Steinbakk et al. 2011) |
| P16 | 2011 | 152 | Both | 11 | 57 Months (Median) | p16, Ready to use, MTM tech | Authors found high expression in EH, rising between EH &EC. They demonstrate >80% p16 expression prognostic in progression of EH to EC. | (Steinbakk et al. 2011) |

EH = Endometrial Hyperplasia, EC = Endometrial Cancer, *WHO = World Health Organisation 2003 (or earlier) classification system for Endometrial Hyperplasia, EIN = Endometrial Intraepithelial Neoplasia classification system for Endometrial Hyperplasia.

†Concurrent EC not diagnosed on initial index biopsy but present in subsequent hysterectomy specimen 12 weeks later.

§Coexisting or subsequent development of EC.
